# Aryl hydrocarbon receptor confers protection against macrophage pyroptosis and intestinal inflammation through regulating polyamine biosynthesis

**DOI:** 10.7150/thno.95749

**Published:** 2024-07-08

**Authors:** Yajing Gao, Kwei-Yan Liu, Wenfeng Xiao, Xueru Xie, Qiuyan Liang, Zikun Tu, Lan Yang, Hongmiao Yu, Haiyan Guo, Saihua Huang, Xiao Han, Jinrong Fu, Yufeng Zhou

**Affiliations:** 1 Department of Critical Care Medicine, Children's Hospital of Fudan University, National Children's Medical Center, and the Shanghai Key Laboratory of Medical Epigenetics, International Co-laboratory of Medical Epigenetics and Metabolism, Ministry of Science and Technology, Institutes of Biomedical Sciences, Fudan University, Shanghai 200032, China.; 2 National Health Commission (NHC) Key Laboratory of Neonatal Diseases, Fudan University, Shanghai, China.; 3 National Institute of Environmental Health Sciences, National Health Research Institutes, Taiwan.

**Keywords:** Aryl hydrocarbon receptor, macrophage, pyroptosis, ODC1, spermine

## Abstract

**Rationale:** The aryl hydrocarbon receptor (AhR) functions in the regulation of intestinal inflammation, but knowledge of the underlying mechanisms in innate immune cells is limited. Here, we investigated the role of AhR in modulating the functions of macrophages in inflammatory bowel disease pathogenesis.

**Methods:** The cellular composition of intestinal lamina propria CD45^+^ leukocytes in a dextran sulfate sodium (DSS)-induced mouse colitis model was determined by single-cell RNA sequencing. Macrophage pyroptosis was quantified by analysis of lactate dehydrogenase release, propidium iodide staining, enzyme-linked immunosorbent assay, western blot, and flow cytometry. Differentially expressed genes were confirmed by RNA-seq, RT-qPCR, luciferase assay, chromatin immunoprecipitation, and immunofluorescence staining.

**Results:** AhR deficiency mediated dynamic remodeling of the cellular composition of intestinal lamina propria (LP) CD45^+^ immune cells in a colitis model, with a significant increase in monocyte-macrophage lineage. Mice with AhR deficiency in myeloid cells developed more severe dextran sulfate sodium induced colitis, with concomitant increased macrophage pyroptosis. Dietary supplementation with an AhR pre-ligand, indole-3-carbinol, conferred protection against colitis while protection failed in mice lacking AhR in myeloid cells. Mechanistically, AhR signaling inhibited macrophage pyroptosis by promoting ornithine decarboxylase 1 (*Odc1*) transcription, to enhance polyamine biosynthesis. The increased polyamine, particularly spermine, inhibited NLRP3 inflammasome assembly and subsequent pyroptosis by suppressing K^+^ efflux. *AHR* expression was positively correlated with *ODC1* in intestinal mucosal biopsies from patients with ulcerative colitis.

**Conclusions:** These findings suggest a functional role for the AhR/ODC1/polyamine axis in maintaining intestinal homeostasis, providing potential targets for treatment of inflammatory bowel disease.

## Introduction

Aryl hydrocarbon receptor (AhR) is a ligand-activated transcription factor that responds to a diverse range of exogenous compounds, such as environmental pollutants, as well as endogenous signals derived from dietary components, microbiota, and cellular metabolites 1. In the absence of ligands, AhR forms a stable complex with Hsp90, XAP2, and p23 in the cytoplasm. Upon ligand binding, AhR translocates from the cytoplasm to the nucleus where it dimerizes with aryl hydrocarbon receptor nuclear translocator, to enhance binding to promoter regions of downstream genes at xenobiotic response elements (XREs) 2. Recent studies have identified cell-intrinsic roles for AhR in the development, differentiation, survival, and maintenance of several immune cell types, highlighting the pivotal function of this molecule in regulating physiological homeostasis and immune responses 3-5; however, the detailed molecular mechanisms through which AhR modulates inflammatory responses remain poorly understood.

Inflammatory bowel disease (IBD), comprised of Crohn's disease (CD) and ulcerative colitis (UC), is an idiopathic and relapsing inflammatory disease of the gastrointestinal tract 6. There is accumulating evidence that environmental, dietary, microbial, and immunological cues regulate intestinal inflammation in mouse models by targeting AhR in epithelial cells 7, 8, innate lymphoid cells (ILCs) 9, 10, intraepithelial lymphocytes 11, and T cells 12-14; however, little is known about the exact role of AhR in regulating intestinal macrophage functions in IBD pathogenesis. To maintain intestinal homeostasis, the gut is equipped with one of the largest populations of macrophages in the body 15. Once pathogens break through the epithelial barrier and invade the intestinal mucosa, they can be recognized by macrophages via pattern recognition receptors, which induce a series of inflammatory responses 16. Overactivated macrophages participate in IBD pathogenesis by controlling the initiation and amplification of local inflammation 17. In response to lipopolysaccharide (LPS), macrophages derived from AhR-null mice exhibited hyper-activated expression of the pro-inflammatory cytokines (e.g., IL-1β, IL-6) 18, 19, whereas production of the anti-inflammatory cytokine, IL-10, was decreased 20. Altered expression of cytokines by macrophages may underlie AhR-mediated regulation of inflammatory responses; however, the mechanisms through which AhR modulates macrophage activation and cytokine release remain to be fully elucidated.

Pyroptosis is a lytic form of programmed cell death, characterized by pore formation in the cell membrane, cell swelling, and ultimate release of large amounts of cell contents, including pro-inflammatory cytokines 21. The main pyroptosis signaling pathway is mediated by inflammasome dependent caspase-1 activation, resulting in a process involving IL-1β, IL-18, and gasdermin D (GSDMD) maturation 22, 23. Dysregulation of pyroptosis is often associated with excessive inflammatory responses, that lead to various inflammatory diseases, including IBD 24, 25. Genome-wide association study analysis has pointed to several IBD susceptibility genes encoding proteins associated with pyroptosis, including IL-1 antagonist receptor (IL-1-RN), IL-1β, IL-18 receptor accessory protein (IL-18RAP), and IL-18 receptor 1 (IL-18R1) 26, 27. Further, large numbers of pro-inflammatory macrophages are reported to infiltrate the gut mucosa of patients with UC and CD 28, 29, while increased levels of pro-inflammatory cytokines, including IL-1β and IL-18, are detected in active IBD and correlate with inflammation severity 2, 30-32.

In the current study, we discovered that, in a colitis mouse model, AhR deficiency mediated dynamic remodeling of the cellular composition of intestinal lamina propria (LP) CD45^+^ immune cells, with a significant increase in the monocyte-macrophage lineage. Significantly, we show that AhR signaling protects against macrophage pyroptosis and intestinal inflammation via the AhR-ODC1-polyamine axis, both *in vitro* and *in vivo*.

## Results

### AhR deficiency exacerbated dextran sulfate sodium (DSS)-induced colitis and LPS-induced inflammation

**Figure 1:**
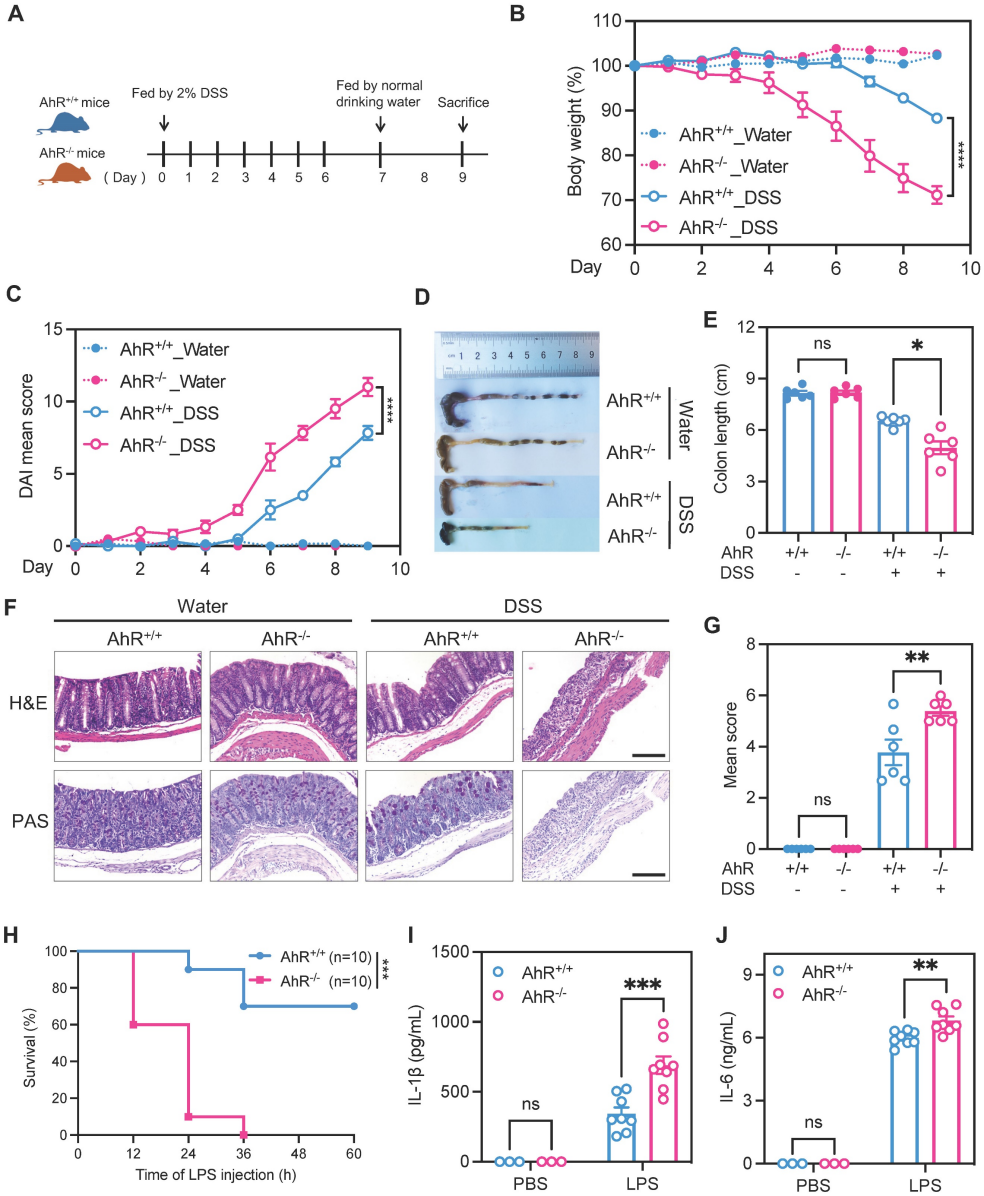
** AhR deficiency exacerbated DSS-induced colitis and LPS-induced inflammation.** (A) Schematic representation of the DSS-induced colitis model. AhR-null (AhR^-/-^) mice and co-housed wide type (AhR^+/+^) littermates were orally administered 2% (w/v) DSS in drinking water for 7 days, followed by regular drinking water for the next 2 days. Mice were sacrificed on day 9. (B) Body weight changes were monitored daily and are presented as percentage of initial body weight (n = 6 per group). (C) DAI scores were determined daily for each AhR^-/-^ and AhR^+/+^ mouse, based on stool consistency, fecal bleeding, and body weight loss (n = 6 per group). (D) Representative morphology images of colon specimens from each group. (E) Colon lengths were measured and recorded on day 9 (n = 6 per group). (F) Representative histopathological images of H&E and PAS-stained colon sections. Scale bar, 200 μm. (G) Semiquantitative histopathology scoring (n = 6 per group). (H) Survival plots for AhR^+/+^ (n = 10) and AhR^-/-^ mice (n = 10) intraperitoneally injected with LPS (20 mg/kg). (I) IL-1β and (J) IL-6 concentrations in serum from AhR^+/+^ and AhR^-/-^ mice 16 h after LPS (10 mg/kg) challenge; n = 8 per group. Statistical analysis of the data was performed using one-way ANOVA with Welch's correct (E) or not (G), two-way ANOVA (B, C, I, J) followed by either Tukey's, Sidak's or Dunnett's T3 multiple comparisons test, and Log-rank (Mantel-Cox) test (H). Data are presented as the mean ± SEM. ns, not significant; *P < 0.05, **P < 0.01, *** P < 0.001, and **** P < 0.0001. AhR, aryl hydrocarbon receptor; DSS: dextran sulfate sodium salt; DAI: disease activity index; H&E, hematoxylin and eosin; PAS, periodic acid-Schiff.

To investigate the potential role of AhR in regulating inflammatory responses *in vivo*, age- and sex-matched AhR-null (AhR^-/-^) mice and co-housed wild type (AhR^+/+^) littermates were orally administered 2% DSS to induce acute colitis, as described previously (Figure 1A) 33. Relative to controls administered with water, DSS-treated mice exhibited typical symptoms of colitis, including diarrhea, hematochezia, and body weight loss. Relative to DSS-treated littermate controls, DSS-treated AhR^-/-^ mice showed more severe colitis, characterized by greater body weight loss, higher disease activity index (DAI) score, and shorter colon length (Figure 1B-E). Histopathological analysis demonstrated that treatment of AhR^-/-^ mice with DSS led to more severe transmural inflammation, with disruption of the mucosal epithelium, focal areas of extensive ulceration, and loss of goblet cells (Figure 1F-G).

An LPS-induced sepsis model, generated by intraperitoneal injection of AhR^+/+^ and AhR^-/-^ mice with LPS (20 mg/kg), was used to confirm the function of AhR in acute inflammation *in vivo*. After 36 h, AhR^+/+^ mice showed 70% survival, whereas all AhR^-/-^ mice had died (Figure 1H). The exacerbated inflammatory responses in LPS-injected AhR^-/-^ mice were further emphasized by marked increases in plasma concentrations of IL-1β and IL-6 (Figure 1I-J).

Collectively, these results indicate that AhR deficiency exacerbated DSS-induced colitis and LPS-induced inflammation, suggesting a protective role for AhR in controlling inflammatory responses and immune homeostasis.

### Single-cell RNA sequencing (scRNA-seq) analysis of LP CD45^+^ cells revealed an increase in the pro-inflammatory monocyte-macrophage lineage in AhR^-/-^ mice during colitis

**Figure 2:**
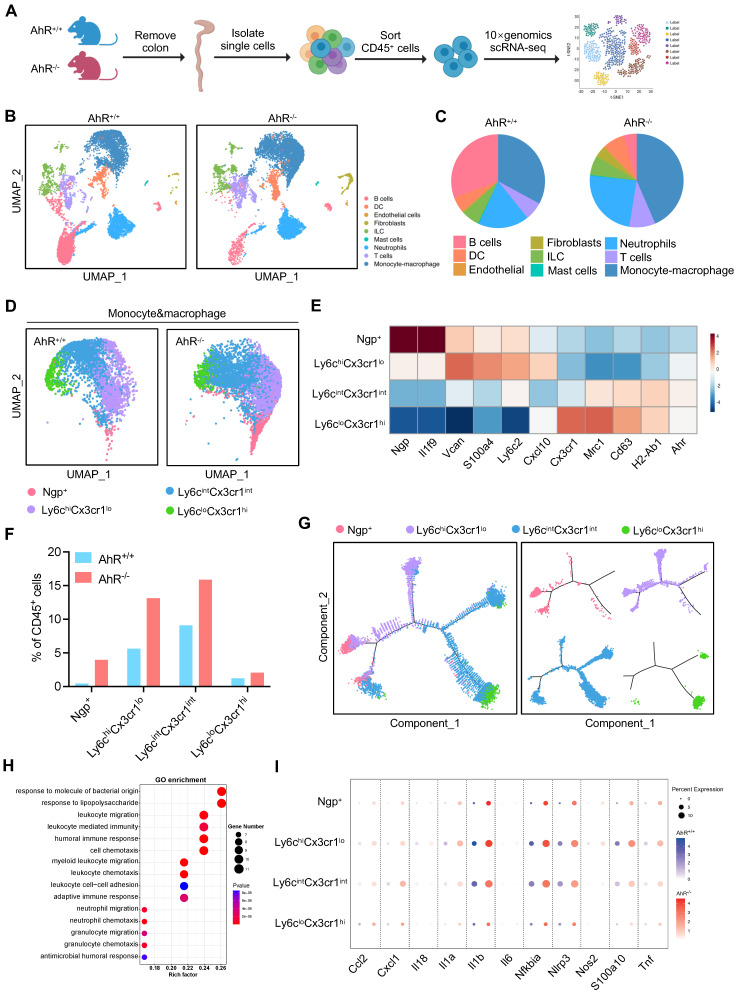
** Distinct transcriptional signatures of LP CD45^+^ immune cells from AhR^-/-^ mice and AhR^+/+^ littermates determined by high-throughput single cell RNA sequencing (scRNA-seq) analysis.** (A) Experimental schematic of scRNA-seq. Briefly, 8-week-old AhR^+/+^ and AhR^-/-^ mice were administered 2% DSS in drinking water for 7 days to induce colitis, and intestinal LP CD45^+^ immune cells isolated for scRNA-seq analysis on day 8 using CD45^+^ immunological microbeads. (B) UMAP and graph visualization of LP CD45^+^ cells (16787 AhR^-/-^ and 20844 AhR^+/+^ cells) defined nine clusters. (C) Pie charts of cell-type fractions for LP CD45^+^ cells from AhR^-/-^ mice and AhR^+/+^ mice, colored according to cell type. (D) UMAP and graph visualization of the four monocyte-macrophage lineage cell subsets detected in the two mouse strains. (E) Heatmap showing normalized expression of representative DEGs in each monocyte-macrophage lineage subset. (F) Proportions of AhR^-/-^ and AhR^+/+^ monocyte-macrophage lineages within each cluster relative to CD45^+^ immune cells. (G) Pseudotime trajectory analysis of the four monocyte-macrophage lineage cell subsets. (H) Gene ontology terms for the most enriched pathways in intestinal monocyte-macrophage lineages from AhR^-/-^ and AhR^+/+^ mice. (I) Dot plots showing representative DEGs between AhR^-/-^ and AhR^+/+^ intestinal monocyte-macrophage lineage cell subsets. AhR, aryl hydrocarbon receptor; DEGs, differentially expressed genes; LP, lamina propria; scRNA-seq, single cell RNA sequence; UMAP, uniform manifold approximation and projection.

Next, we sought to identify potential effector cells contributing to the exacerbated inflammatory responses in AhR^-/-^ mice. Recent studies highlight the essential role of AhR in various immune cell types during IBD. Hence, a comprehensive and unbiased approach with single-cell resolution to investigate AhR functions in immune cells is essential for understanding the pathogenesis of IBD. Therefore, we conducted scRNA-seq to analyze intestinal LP CD45^+^ leukocytes from the colonic tissue of AhR^+/+^ and AhR^-/-^ mice with colitis (Figure 2A). We purified 37613 high-quality LP CD45^+^ leukocytes, including 16787 from AhR^-/-^ mice and 20844 from AhR^+/+^ littermates. The transcriptomic diversity of the resulting data was projected onto two dimensions by uniform manifold approximation and projection (UMAP). Unsupervised clustering analysis identified nine distinct cell types, which were annotated based on known lineage markers, including B cells, dendritic cells (DCs), ILCs, mast cells, neutrophils, T cells, and monocyte-macrophage lineage (Figure 2B, Figure S1A-C). The proportion of B cells relative to CD45^+^ immune cells was lower in AhR^-/-^ mice than in AhR^+/+^ mice, while those of DCs, ILCs, and T cells were comparable (Figure 2C). Myeloid cells, including monocyte-macrophage lineage and neutrophils, were predominate in the colonic LP. Notably, 38.5% of LP CD45^+^ cells were intestinal monocyte-macrophage lineage in AhR^-/-^ mice, compared with only 22.1% in AhR^+/+^ controls (Figure 2C). scRNA-seq transcription profiles showed that, relative to monocyte-macrophage lineage, neutrophils exhibited minimal AhR expression (Figure S1D). Since the monocyte-macrophage lineage are the most abundant cells in the LP, and infiltrating macrophages are known to produce pro-inflammatory cytokines resulting in local uncontrolled inflammation during IBD, we next analyzed the heterogeneity of the intestinal monocyte-macrophage pool. UMAP clustering was applied to divide the monocyte-macrophage lineage into four distinct clusters, based on their respective transcriptional signatures (Figure 2D-E). Among these clusters, Ngp^+^ monocytes exhibited high expression of *Ngp* and *Il1f9*, and were identified as newly-recruited monocytes; Ly6c^hi^Cx3cr1^lo^ monocytes exhibited high expression of *Vcan*, *Ly6c2*, *S100a4*, and *Cxcl10*, similar to hemopoietic system-derived classical inflammatory monocytes; Ly6c^int^Cx3cr1^int^ macrophages exhibited high expression of major histocompatibility complex class II (MHC-II) genes (e.g., *H2-Aa*, *H2-Ab1*, *H2-DMb1*, and *H2-Eb1*), and were identified as inflammation-elicited plastic macrophages; and Ly6c^lo^Cx3cr1^hi^ macrophages expressed high levels of *Cx3cr1*, *Cd163*, *Mrc1*, and *Tgfbr2*, similar to tissue resident macrophages (Figure 2E, Figure S2A-B). Moreover, the proportions of Ngp^+^ monocytes, Ly6c^hi^Cx3cr1^lo^ monocytes, and Ly6c^int^Cx3cr1^int^ macrophages were significantly higher in the LP of AhR^-/-^ mice than those in AhR^+/+^ controls, whereas the proportion of tissue resident macrophages (Ly6c^lo^Cx3cr1^hi^) was comparable in both mouse groups (Figure 2F). Further, a pseudotime-organized sequence of “differentiation/activation” events showed that recruited monocytes (Ngp^+^ and Ly6c^hi^Cx3cr1^lo^ monocytes) differentiate into tissue resident macrophages (Ly6c^lo^Cx3cr1^hi^). During intestinal inflammation, this differentiation process is disrupted, leading to accumulation of Ly6c^int^Cx3cr1^int^ macrophages, which produce inflammatory mediators (Figure 2G). Notably, gene ontology enrichment analysis demonstrated that enriched genes were highly associated with the response to the LPS pathway in monocyte-macrophage lineage of AhR^-/-^ mice (Figure 2H). Moreover, we observed that the expression levels of inflammatory cytokines (e.g., *Il1β*, *Il1α*, *Il18*, *Il6*,* Tnf*), chemokines (e.g., *Cxcl1*), and antibacterial peptides (e.g., *S100a10*) were significantly higher in Ngp^+^, Ly6c^hi^Cx3cr1^lo^ and Ly6c^int^Cx3cr1^int^ monocyte-macrophage subsets from AhR^-/-^ mice than those in AhR^+/+^ mice (Figure 2I). These results suggest that AhR deletion leads to an increased accumulation of monocyte-macrophage lineage in the gut microenvironment, concomitant with enhanced inflammatory responses.

### AhR deficiency in macrophages exacerbated DSS-induced colitis

To investigate AhR functions in modulating the role of macrophages in IBD pathogenesis, we generated mice with myeloid cell-specific knock-out of AhR (AhR^fl/fl^;LysM-Cre^+^, hereafter AhR^△Mye^ mice) by crossing lysozyme-cre mice with AhR^fl/fl^ mice (Figure S3A-B); co-housed littermates AhR^fl/fl^ mice were used as controls. The efficiency of AhR knockout in macrophages was confirmed by western blot analysis of bone marrow derived macrophages (BMDMs) (Figure S3C).

**Figure 3:**
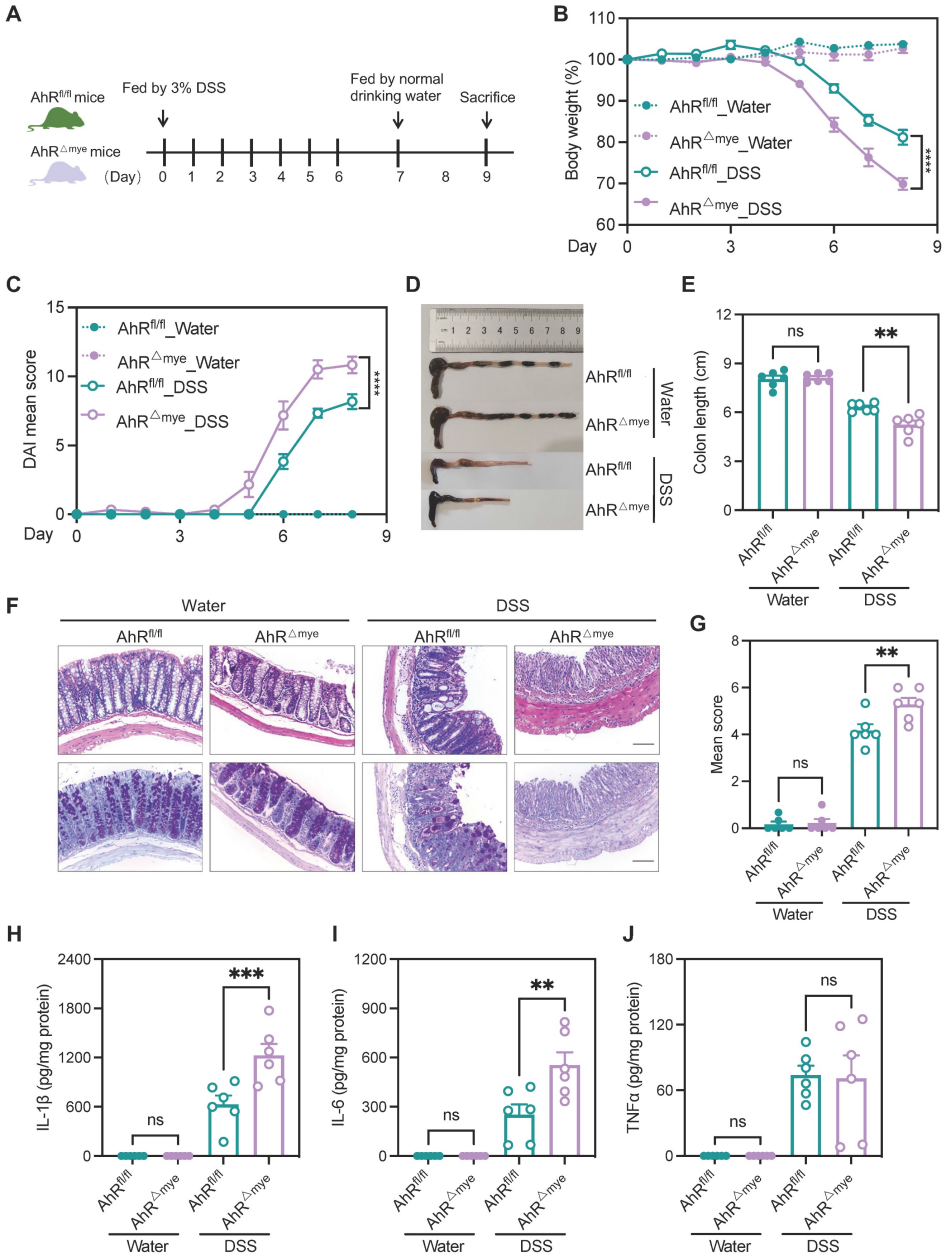
** AhR deficiency in macrophages exacerbated DSS-induced colitis.** (A) Schematic representation of DSS-induced colitis model. AhR^△Mye^ mice and co-housed AhR^fl/fl^ littermates were administered 3% (w/v) DSS in drinking water for 7 days, followed by regular drinking water for 1 day. Mice were sacrificed on day 8. (B) Body weight changes were monitored daily and are presented as a percentage of initial body weight (n = 6 per group). (C) DAI was scored daily for each AhR^△Mye^ and AhR^fl/fl^ mouse based on stool consistency, fecal bleeding, and body weight loss (n = 6 per group). (D) Representative morphology images of colon specimens from each group. (E) Colon lengths were measured and recorded on day 8 (n = 6 per group). (F) Representative histopathological images of H&E and PAS-stained colon sections. Scale bar, 200 μm. (G) Semiquantitative scoring of histopathology. (H-J) Expression levels of IL-1β, IL-6, and TNFα in colon tissue quantified by ELISA; n = 6 per group. Statistical analysis of the data was performed using one-way ANOVA (E, G, H, I, J), and two-way ANOVA (B, C) followed by Tukey's multiple comparisons test. Data are presented as the mean ± SEM. ns, not significant; **P < 0.01, *** P < 0.001, and **** P < 0.0001. AhR, aryl hydrocarbon receptor; DSS: dextran sulfate sodium salt; DAI: disease activity index; H&E, hematoxylin and eosin; PAS, periodic acid-Schiff.

These cells failed to induce expression of the AhR target gene, *Cyp1a1,* on stimulation with the AhR ligands, 6-Formylindolo[3,2-b] carbazole (FICZ) or indeno[1,2,3-*cd*]pyrene (IP) (Figure S3D), confirming efficient and specific AhR deletion in macrophages. Additionally, no apparent abnormalities of external morphology were noted in AhR^△Mye^ mice relative to AhR^fl/fl^ mice. Gut microbiota balance is critical for intestinal homeostasis regulation; however, 16s rDNA analysis revealed that microbial community diversity and phylum composition were comparable between 8-week-old AhR^△Mye^ mice and their AhR^fl/fl^ littermates (Figure S4A-C). Next, age- and sex-matched AhR^△Mye^ mice and co-housed AhR^fl/fl^ littermates were orally administered 3% DSS to induce acute colitis (Figure 3A). Relative to controls receiving water, DSS-treated mice exhibited typical symptoms of colitis, with diarrhea, hematochezia, and body weight loss. Further, DSS-treated AhR^△Mye^ mice showed significantly greater body weight loss, and had higher DAI scores and shorter colons than DSS-treated AhR^fl/fl^ littermates (Figure 3B-E). Histopathological analysis showed that treatment of AhR^△Mye^ mice with DSS led to more severe transmural inflammation, with disruption of the mucosal epithelium, focal areas of extensive ulceration, and more extensive goblet cell loss (Figure 3F-G). Moreover, levels of pro-inflammatory cytokines (e.g., IL-1β and IL-6) were higher in colonic tissue from DSS-treated AhR^△Mye^ mice than those in AhR^fl/fl^ littermates, while TNFα levels did not differ significantly between these two groups (Figure 3H-J).

**Figure 4:**
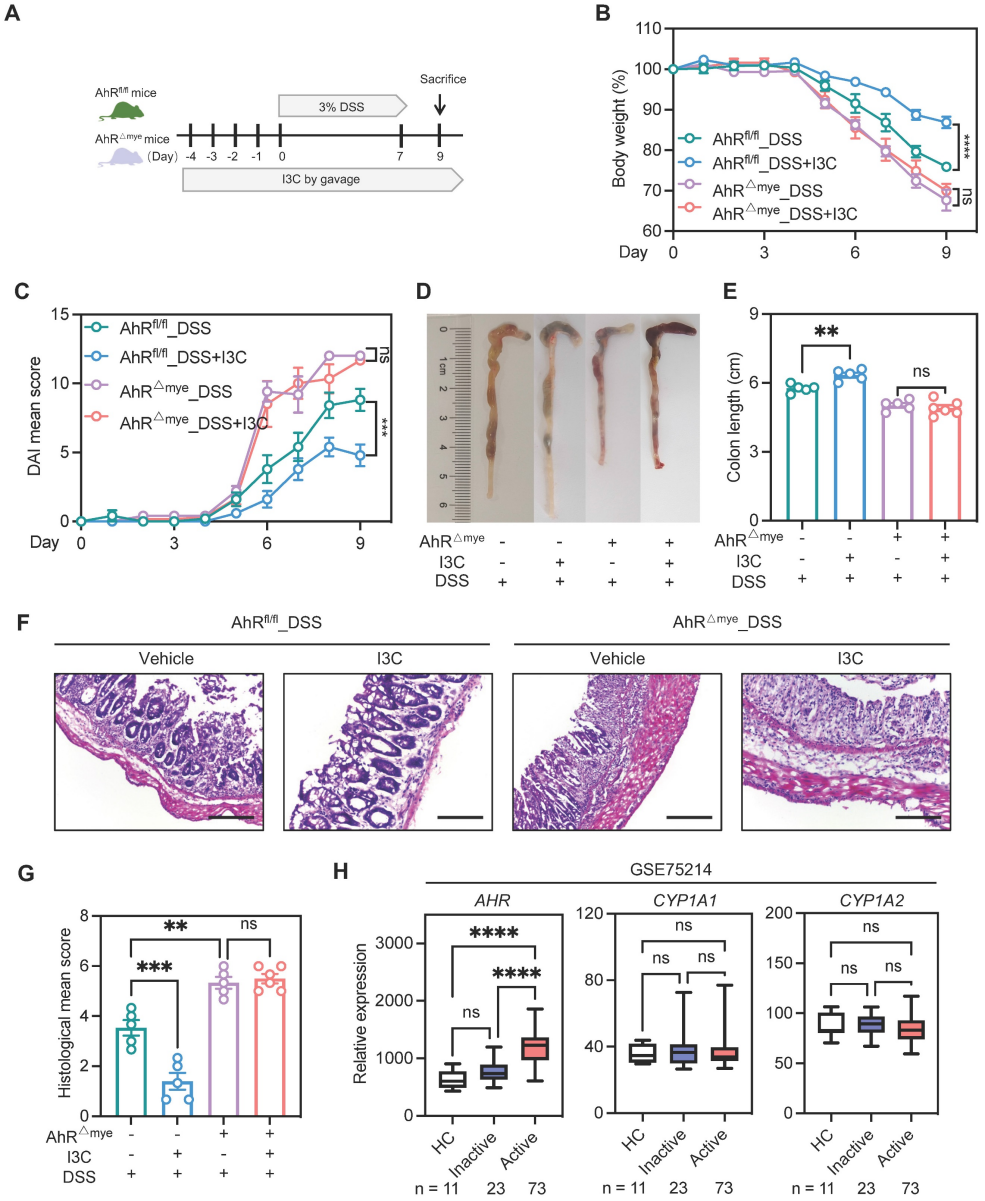
** Pharmacologic activation of AhR alleviated inflammation in the DSS-induced colitis model, dependent on macrophages.** (A) Schematic illustration of DSS-induced colitis. Age- and sex-matched AhR^△Mye^ and AhR^fl/fl^ mice were administered 3% DSS in drinking water for 7 days followed by normal drinking water for 2 days. Mice in I3C treatment groups received daily gavage of I3C for 14 days. (B) Body weight changes were monitored daily and are depicted as a percentage of initial body weight. (C) DAI was scored daily for each mouse, based on stool consistency, fecal bleeding, and body weight loss. (D) Representative images showing the morphology of colons from each group. (E) Colon lengths were measured and recorded on day 9. (F) Representative histopathological images of H&E-stained colon sections. Scale bar, 200 μm. (G) Semiquantitative scoring of histopathology. n = 5-6 per group. (H) Database (GSE75214) analysis of *AHR*, *CYP1A1*, and *CYP1A2* mRNA in intestinal mucosal biopsies from patients with UC and healthy controls. Statistical analysis of the data was performed using one-way ANOVA (E, G, H *CYP1A2*) and two-way ANOVA (B, C) followed by Tukey's multiple comparisons test, or Welch ANOVA tests followed by Dunnett's T3 multiple comparisons test (H *AHR*), or Kruskal-Wallis test followd by Dunn's multiple comparisons test (H *CYP1A1*). Data are shown as the mean ± SEM. ns, not significant; **P < 0.01, ***P < 0.001, and ****P < 0.0001. AhR, aryl hydrocarbon receptor; DSS: dextran sulfate sodium salt; DAI: disease activity index; H&E, hematoxylin and eosin; HC, healthy control; I3C, indole-3-carbinol; UC, ulcerative colitis.

Pharmacologic activation of AhR is reported to rescue the intestinal inflammatory phenotype 34. Therefore, to further confirm the contribution of macrophage AhR in colitis, we treated AhR^△Mye^ mice and AhR^fl/fl^ mice with indole-3-carbinol (I3C), a plant-derived AhR ligand precursor, by daily oral gavage beginning from 4 days before DSS treatment until the end of the experiments (Figure S5A). Mice treated with I3C showed AhR activation, evidenced by the induction of the AhR target gene, *Cyp1a1*, in colonic tissue (Figure S5B). Administration of I3C alone had no obvious impact on mouse body weight, colon length, or colonic structure (Figure S5C-F). Next, mice were treated with I3C and DSS (Figure 4A); AhR^fl/fl^ mice treated with I3C effectively ameliorated the colitis disease symptoms, with reduced body weight loss, lower DAI score, and extended colon length compared with the DSS group (Figure 4B-E). The beneficial effects mediated by I3C were also confirmed by histopathology scores (Figure 4F-G). In contrast, AhR^△Mye^ mice treated with I3C did not exhibit colonic inflammation rescue, suggesting that the effect of I3C is partially mediated through targeting AhR in macrophages (Figure 4B-G). Further, we analyzed transcriptome profiles from independent cohorts of patients with UC (GSE75214) and found that *AHR* mRNA levels were elevated in active UC mucosa relative to those in healthy controls, while no significant difference was detected in the AhR target gene, *CYP1A1* and *CYP1A2* (Figure 4H). Collectively, these data suggest that macrophage-specific AhR activation is required to confer protection from DSS-induced colonic damage and colitis.

### AhR deficiency enhanced IL-1β secretion by promoting macrophage pyroptosis

**Figure 5:**
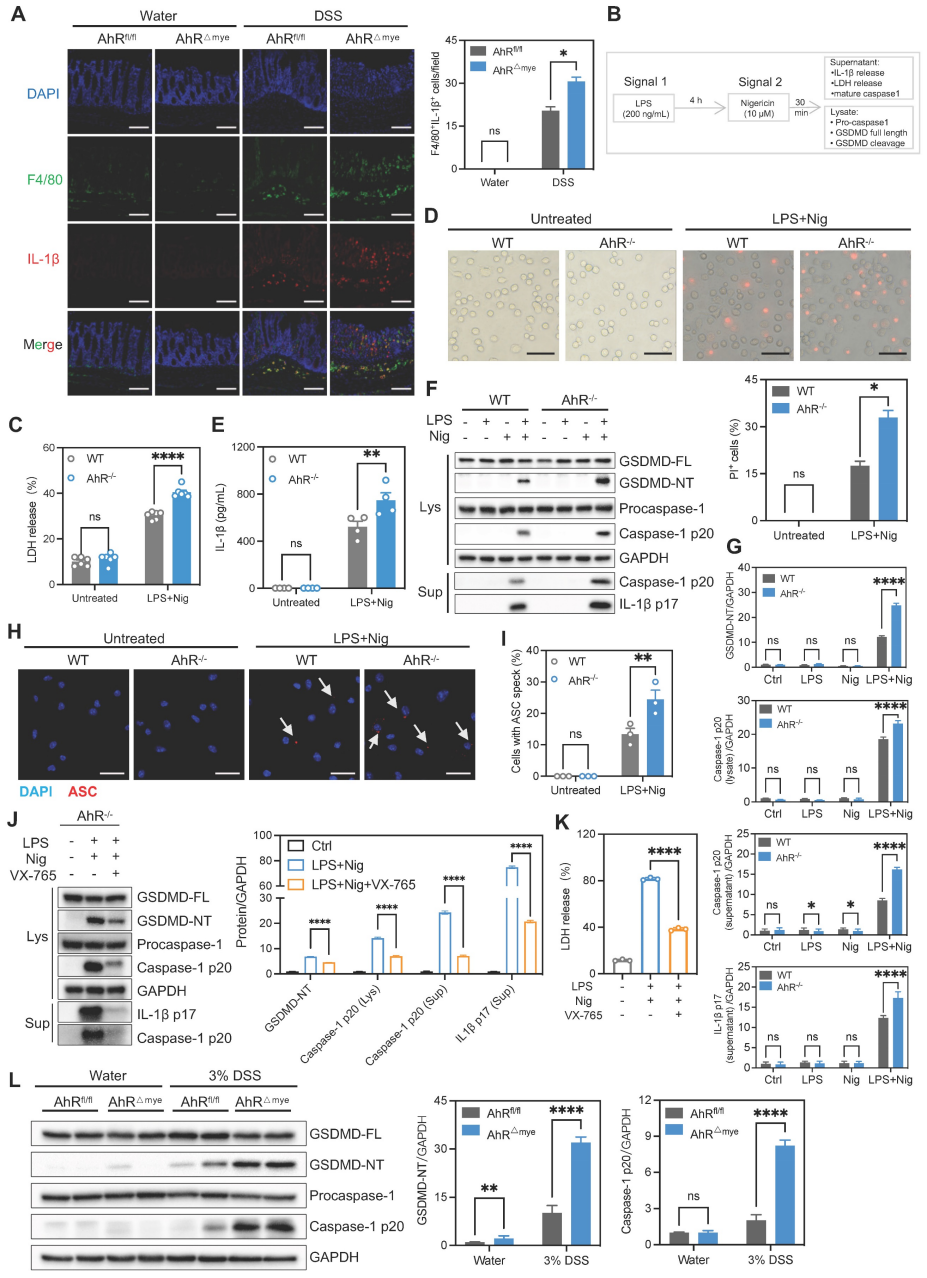
**AhR deficiency enhanced IL-1β secretion by promoting macrophage pyroptosis.** (A) Immunofluorescence labeling of IL-1β (red), F4/80^+^ (green) macrophages, and DAPI (blue) in colon sections from control or DSS-treated AhR^△Mye^ and AhR^fl/fl^ mice. Scale bar, 50 μm. (B-I) BMDMs isolated from WT or AhR knockout (AhR^-/-^) mice were stimulated with LPS (200 ng/mL) for 4 h, followed by treatment with Nig for 45 min. (B) Schematic of experimental design. (C) Cytotoxicity was assessed by LDH release. (D) Cell morphology assessed by PI staining. Scale bar, 50 μm. (E) IL-1β levels in cell culture supernatants determined by ELISA. (F) Cell lysates and supernatants were immunoblotted to detect full-length and cleaved caspase-1 and GSDMD, and mature IL-1β (IL-1β p17). (G) Quantitative protein analysis for F. (H) Representative immunofluorescence images of ASC speck formation (red). Nuclei were stained with DAPI (blue). Scale bar, 20 μm. (I) Bar chart showing percentages of BMDMs containing a visible ASC speck. (J-K) AhR^-/-^ BMDMs were pretreated with VX-765 (50 μM) or vehicle control for 30 min, then stimulated with LPS for 4 h followed by Nig for 45 min. (J) Proteins from cell lysates and supernatants were immunoblotted for full-length and cleaved caspase-1 and GSDMD, and mature IL-1β (IL-1β p17). (K) Cytotoxicity was assessed by LDH release. (L) Immunoblot analysis of full-length and cleaved GSDMD and caspase-1 in colon tissues from control or DSS-treated AhR^△Mye^ and AhR^fl/fl^ mice. n = 3-6 per group. Statistical analysis of the data was performed using one-way ANOVA (K) and two-way ANOVA (A, C, D, E, G, I, J, L) followed by either Tukey's or Sidak's multiple comparison tests. Data are shown as the mean ± SEM from three independent experiments. ns, not significant; *P < 0.05, **P < 0.01, and ****P< 0.0001. AhR, aryl hydrocarbon receptor; ASC, apoptosis associated speck like protein containing a CARD; BMDM, bone barrow derived macrophage; LDH, lactate dehydrogenase; Nig, nigericin; PI, propidium iodide; WT, wild type.

We detected increased numbers of F4/80-labeled macrophages in the DSS group, suggesting increased macrophages that resided in and were recruited to the colon (Figure 5A). Infiltrating macrophages are known to produce pro-inflammatory cytokines, resulting in local uncontrolled inflammation during IBD 29. Therefore, we examined whether AhR deficiency disturbed cytokine expression. The results showed the increased presence of F4/80^+^IL-1β^+^ macrophages infiltrating in DSS-treated AhR^△Mye^ mice relative to AhR^fl/fl^ mice (Figure 5A), consistent with increased IL-1β expression in colon tissues (Figure 3H). IL-1β is a secreted effector protein produced by activation of the NLRP3 inflammasome, which is dominantly released by macrophages undergoing pyroptosis 22. As AhR deficiency significantly increased IL-1β levels, we hypothesized that AhR regulates macrophage pyroptosis and cytokine production in the context of NLRP3 activation. Thus, BMDMs from WT or AhR^-/-^ mice were primed with LPS before treatment with the commonly used activator, nigericin (Nig) or ATP (Figure 5B). Pyroptosis, as measured by lactate dehydrogenase (LDH) release, was significantly increased in primary AhR^-/-^ BMDMs relative to WT BMDMs (Figure 5C, Figure S6A). In addition, more AhR^-/-^ BMDMs were positive for staining with propidium iodide (PI; a dead cell dye indicator), indicating more cell death among AhR^-/-^ BMDMs (Figure 5D). NLRP3 inflammasome mediated pyroptosis also leads to caspase-1 activation and GSDMD cleavage, accompanied by secretion of mature IL-1β. As expected, AhR deficiency promoted caspase-1 activation (caspase1-p20), GSDMD cleavage (GSDMD-NT), and mature IL-1β secretion (IL-1β p17) in LPS-primed macrophages treated with Nig or ATP (Figure 5E-G, Figure S6B).

To validate our findings indicating that AhR is involved in NLRP3 inflammasome activation, we assessed speck formation by apoptosis associated speck like protein containing a CARD (ASC), as an indicator of inflammasome assembly. We found that LPS plus Nig-induced ASC speck formation was enhanced in AhR-deficient cells (Figure 5H-I). Total ASC protein levels were not altered (Figure S6C). Moreover, treatment with the caspase-1 inhibitor, VX-765, markedly restored LDH release and IL-1β production in AhR-deficient cells, confirming that cell death induction was indeed caspase-1-dependent (Figure 5J-K). Next, we evaluated *in vivo* pyroptosis activation in both control and DSS-treated mice. Immunoblotting analysis showed substantially increased caspase-1 p20 and GSDMD-NT in colonic tissues from colitis mice relative to water control mice. Further, the up-regulation of caspase-1 p20 and GSDMD-NT was more aggravated in DSS-treated AhR^△Mye^ than in AhR^fl/fl^ mice (Figure 5L). Additionally, mRNA expression levels of pyroptosis-associated genes, including *NLRP3*, *Caspase-1*, *GSDMD*, and *IL-1β* were elevated in active UC mucosa compared with healthy controls (GSE75214) (Figure S6D). Together with our previous results, these findings suggest that AhR deficiency in macrophages promoted macrophage pyroptosis and enhanced IL-1β secretion and may be associated with IBD severity.

### AhR suppressed IL-1β production by promoting *Odc1* transcription

**Figure 6:**
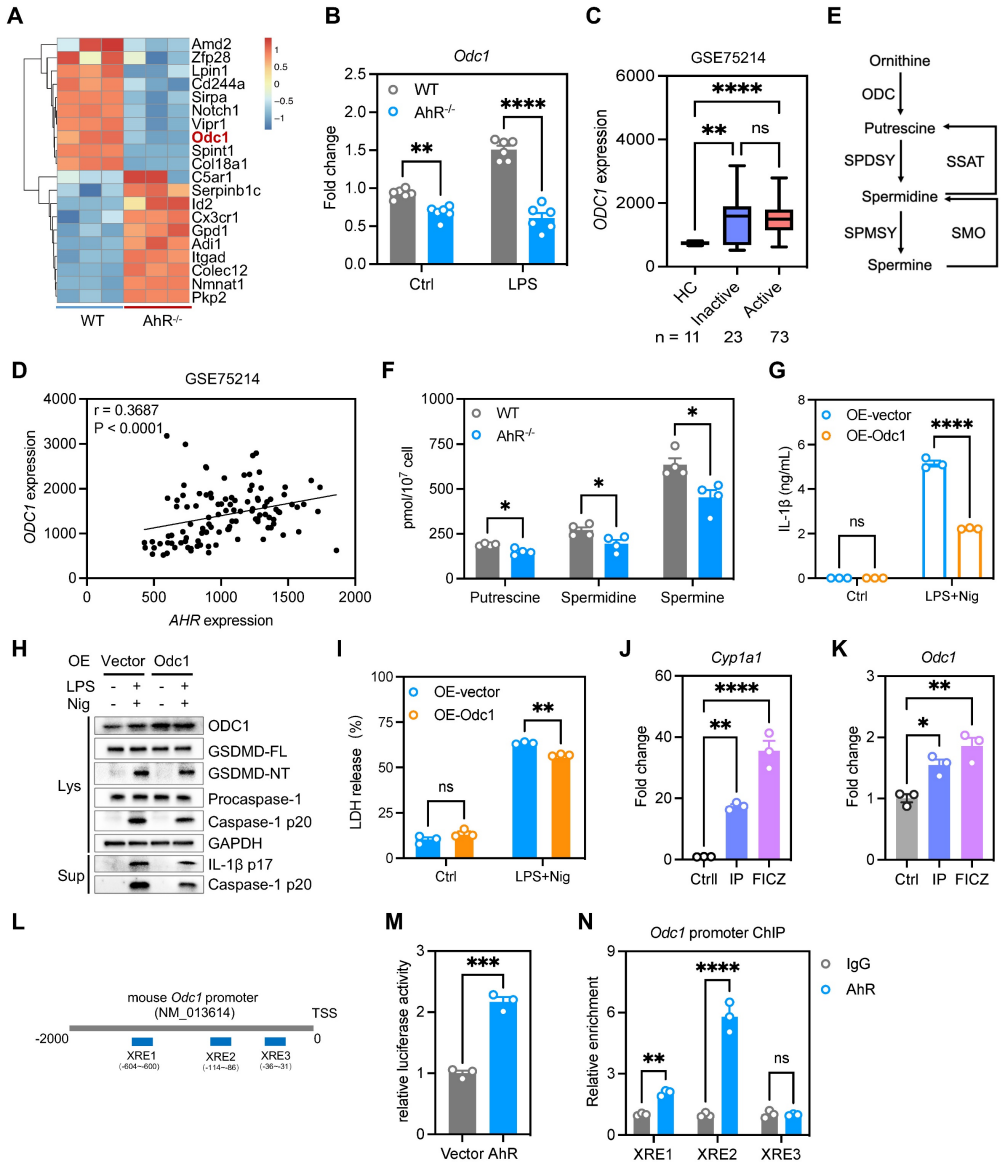
** AhR suppressed IL-1β production by promoting *Odc1* transcription.** (A) Heatmap illustrating differentially expressed genes between WT and AhR^-/-^ BMDMs. (B) *Odc1* expression levels in WT and AhR^-/-^ BMDMs determined by RT-qPCR. (C) Database (GSE75214) analysis of *ODC1* mRNA expression in intestinal mucosal biopsies from patients with UC and healthy controls. (D) Spearman correlation (two-tailed) analysis between *AHR* and *ODC1* expression levels in intestinal mucosal biopsies (GSE75214). (E) Schematic illustration of polyamine biosynthesis. (F) Intracellular polyamine levels measured by liquid chromatography-mass spectrometry. (G) AhR^-/-^ BMDMs were transfected with empty vector control or pcDNA3.1-ODC1 (500 ng) and IL-1β release measured by ELISA. (H) Proteins from cell lysates and supernatants were immunoblotted for full-length and cleaved caspase-1 and GSDMD, and mature IL-1β (IL-1β p17). (I) Cytotoxicity was assessed by LDH release. (J and K) WT BMDMs were pretreated with DMSO or AhR ligands (IP or FICZ) for 24 h, and mRNA expression levels of* Odc1* and the AhR target gene, *Cyp1a1*, measured by RT-qPCR. (L) Schematic diagram of AhR binding sites in the* Odc1* promoter region. (M) Luciferase reporter assay in HEK293T cells co-transfected with Renilla, pGL3-basic-ODC1 promoter (-2000 to +200) reporter, pEGFP-C3 empty vector, or pEGFP-C3-AhR. Values are presented as means, normalized to transfection efficiency by dividing firefly luciferase activity by Renilla luciferase activity. (N) ChIP analyses of the binding efficiency of AhR to the *Odc1* promoter region in RAW264.7 cells. n = 3-6 per group. Statistical analysis of the data was performed using two-tailed unpaired *t* test (M), one-way ANOVA (J, K) and two-way ANOVA (B, F, G, I, N) followed by either Tukey's or Sidak's multiple comparison tests, or Kruskal-Wallis test (C) followed by Dunn's multiple comparisons test. Data are shown as the mean ± SEM from three independent experiments. ns, not significant; *P < 0.05, **P < 0.01, ***P < 0.001, and ****P < 0.0001. AhR, aryl hydrocarbon receptor; BMDM, bone barrow derived macrophage; ChIP, chromatin immunoprecipitation; ELISA, enzyme-linked immunosorbent assay; FICZ, 6-Formylindolo[3,2-b]carbazole; HC, healthy control; IP, indeno[1,2,3-*cd*]pyrene; LDH, lactate dehydrogenase; Nig, nigericin; ODC1, ornithine decarboxylase 1; PI, propidium iodide; RT-qPCR, quantitative reverse transcription polymerase chain reaction; UC, ulcerative colitis; WT, wild type; XRE, xenobiotic response element.

We next explored how AhR regulates the inflammasome to suppress IL-1β production. As IL-1β production is regulated transcriptionally and post-transcriptionally, we first examined the effect of AhR on IL-1β expression; however, IL-1β mRNA or protein levels were not differed significantly between WT and AhR^-/-^ BMDMs (Figure S6E-F). Since AhR deficiency promoted mature IL-1β production, we speculated that the increase in IL-1β production by AhR-deficient cells was due to enhanced secretion, rather than increased synthesis, of IL-1β. We found that AhR did not interact directly with NLRP3 inflammasome components (Figure S6G). Since AhR is a ligand-dependent transcription factor, we next performed RNA-seq of WT and AhR^-/-^ BMDMs, to comprehensively investigate AhR-dependent changes in gene expression associated with IL-1β secretion. Among the genes differentially expressed in AhR^-/-^ macrophages, we noted markedly reduced expression of ornithine decarboxylase 1 (*Odc1*) which is reported to negatively regulate M1 macrophage activation and IL-1β production 35 (Figure 6A, Figure S7A). Reduced *Odc1* expression was confirmed by real-time PCR and western blot analysis in WT and AhR^-/-^ BMDMs (Figure 6B, Figure S7B), as well as in colon tissues in the context of DSS-induced colitis (Figure S7C-E). In addition, we found that *ODC1* mRNA levels were elevated in active UC mucosa relative to those in healthy controls, and that *AHR* expression levels were positively correlated with those of *ODC1* (GSE75214) (Figure 6C-D). As the rate-limiting enzyme in polyamine biosynthesis, ODC1 converts L-ornithine to putrescine, which is subsequentially converted to spermidine and spermine by spermidine and spermine synthases, respectively (Figure 6E). Importantly, AhR depletion resulted in decreased levels of the three major mammalian polyamines, putrescine, spermidine, and spermine, in BMDMs (Figure 6F). Furthermore, we assessed the polyamine levels in colon tissues from DSS-treated AhR^-/-^ and AhR^+/+^ mice. The results showed that, upon DSS treatment, the levels of putrescine, spermidine and spermine were reduced in the colon of AhR^-/-^ mice compared to AhR^+/+^ mice (Figure S7F-H).

To investigate whether the increased IL-1β secretion in AhR^-/-^ BMDMs was due to their reduced *Odc1* expression, we next over-expressed *Odc1* in AhR^-/-^ BMDMs. *Odc1* overexpression significantly inhibited LPS plus Nig-induced secretion of IL-1β compared with empty vector controls (Figure 6G), accompanied by decreased caspase-1 p20 and GSDMD-NT levels, suggesting that decreased *Odc1* expression, at least in part, contributed to the increased IL-1β secretion in AhR-null BMDMs on NLRP3 inflammasome activation (Figure 6H, Figure S7I). Moreover, *Odc1* overexpression inhibited AhR^-/-^ BMDMs pyroptosis, as evidenced by reduced LDH release (Figure 6I). In contrast, *Odc1* depletion promoted caspase-1 p20 activation and GSDMD cleavage, along with increasing IL-1β secretion and LDH release (Figure S8A-D). We also detected the pyroptosis levels in macrophages with ODC1 knockdown, followed by AhR agonist (FICZ) administration in the presence of LPS plus Nig treatment. The results showed that ODC1 knockdown enhanced macrophage pyroptosis, as evidenced by increased activation of pyroptosis-related proteins and LDH release. AhR agonist administration inhibited macrophage pyroptosis, whereas the inhibitory effect was abrogated with ODC1 knockdown (Figure S8E-F). Furthermore, treatment with endogenous (FICZ) and exogenous (IP) AhR ligands induced mRNA expression of *Odc1*, and that of a specific AhR target, *Cyp1a1*, which served as a positive control (Figure 6J-K).

AhR is reported to control gene transcription activity by direct binding to XRE in promoter regions in its target genes 36; therefore, we next determined whether AhR bound to the *Odc1* promoter and modulated its transcription activity. Three putative XREs were identified in the mouse *Odc1* promoter: XRE-1 (nt -604 to -600), XRE-2 (nt -114 to -86), and XRE-3 (nt -35 to -31) (Figure 6L). To assess the function of these XREs in AhR-induced* Odc1* expression, *Odc1* promoter regions containing XREs (nt -2000 to +200) were sub-cloned into the pGL3-basic luciferase reporter plasmid. HEK293T cells transfected, in parallel, with the *Odc1* promoter reporter construct were co-transfected with either AhR expression vector or empty vector as a control. AhR expression significantly increased luciferase reporter activity of *Odc1* promoter (Figure 6M). Next, we performed chromatin immunoprecipitation (ChIP) assays using a commercially available antibody against AhR and primers flanking the putative AhR binding sites. Significant DNA enrichment of XRE1 and XRE2, but not XRE3, was detected in AhR versus IgG-precipitated material (Figure 6N). These data indicate that AhR controls *Odc1* transcription and that decreased *Odc1* expression is at least one of the causes for the increased IL-1β secretion detected in AhR-null BMDMs.

### Spermine prevented macrophage pyroptosis and IL-1β production

**Figure 7:**
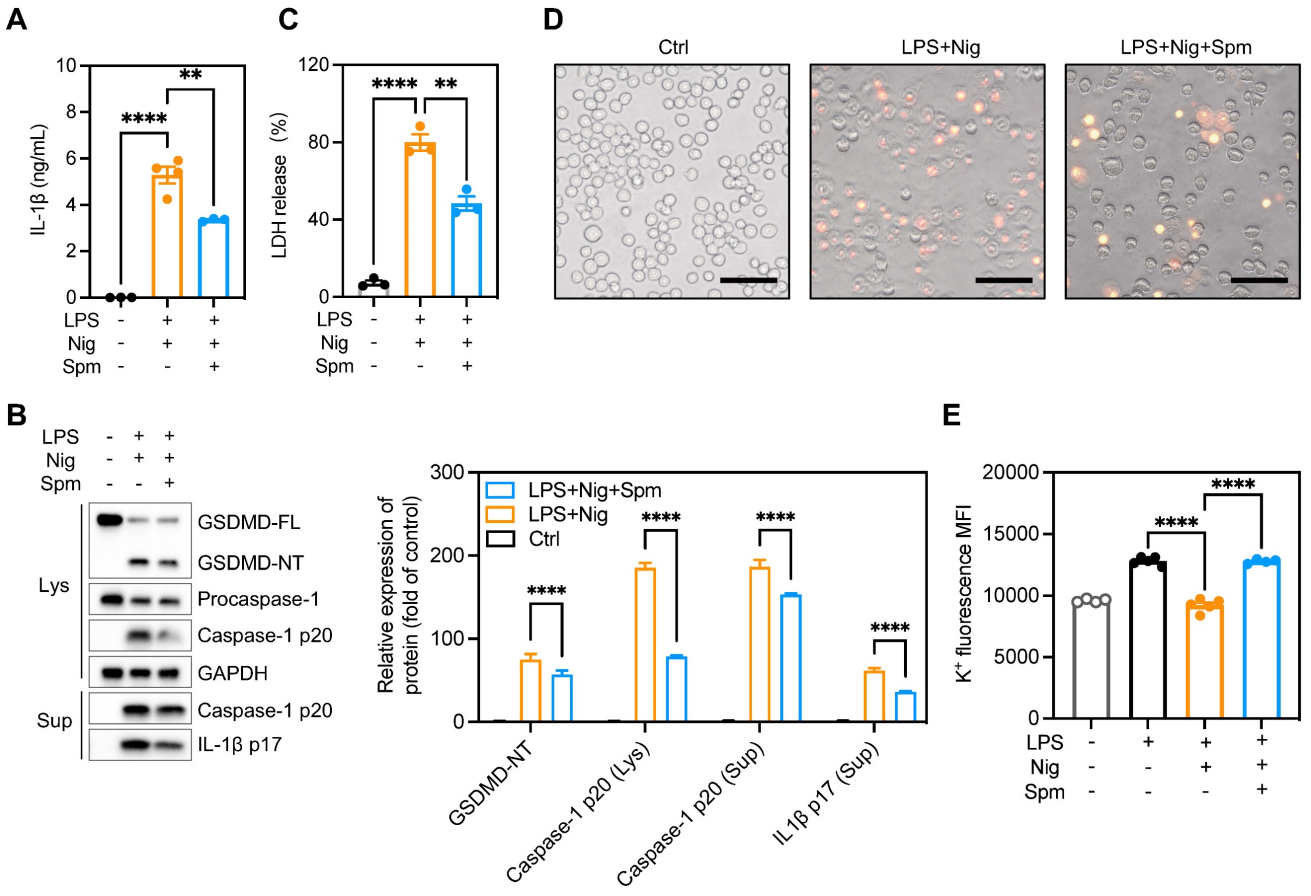
** Spermine prevented macrophage pyroptosis and IL-1β production.** Cells were pretreated with spermine (50 μM) together with LPS (200 ng/mL) for 4 h, then stimulated with nigericin (10 mM) for 30-45 min. (A) IL-1β secretion in supernatants determined by ELISA. (B) Proteins in cell lysates and supernatants were immunoblotted to detect full-length and cleaved caspase-1, GSDMD, and IL-1β. (C) Cytotoxicity was detected by LDH release assay. (D) Cell morphology determined by PI staining. Scale bar, 50 μm. (E) Relative intracellular K^+^ concentration detected by K^+^ ion fluorescence probe; data are shown as MFI. Statistical analysis of the data was performed using one-way ANOVA (A, C, E) and two-way ANOVA (B) followed by Tukey's multiple comparison tests. Data are shown as the mean ± SEM from three independent experiments. **P < 0.01, and ****P < 0.0001. ELISA, enzyme-linked immunosorbent assay; LDH, lactate dehydrogenase; MFI, mean fluorescence intensity; PI, propidium iodide; Spm, spermine.

ODC1 is the first and rate-limiting enzyme in polyamine biosynthesis, with spermine the final product of polyamine metabolism 37. It is unclear whether polyamines regulate macrophage pyroptosis. We hypothesized that addition of exogenous spermine to cell cultures would reverse the enhanced pyroptosis and IL-1β production observed. To test this hypothesis, we treated BMDMs with difluoromethylornithine (DFMO), an irreversible and specific ODC inhibitor, for 3 days and then added exogenous spermine to the culture. The addition of spermine indeed reversed LDH release in DFMO-treated cells (Figure S9A), implying that the effects of ODC1 in inflammation may depend on polyamine biosynthesis pathways. Moreover, exogenous spermine inhibited GSDMD cleavage and abrogated caspase-1 p20 and IL-1β secretion in macrophages undergoing pyroptosis (Figures 7A-B). In addition, exogenous spermine inhibited pyroptosis-induced LDH release (Figure 7C) and reduced the proportion of PI-positive cells (Figure 7D, Figure S9B).

The main pathways related to NLRP3 inflammasome activation include: K^+^ efflux, mitochondria dysfunction, reactive oxygen species (ROS) production and lysosomal rupture 38. Previous studies have demonstrated that polyamines such as spermidine and spermine block K^+^ efflux from cells by voltage-dependent binding to inwardly rectifying K^+^ (Kir) channels and displacing K^+^ ions from the pore 39, 40. To investigate the role of spermine on K^+^ efflux, we analyzed relative intracellular K^+^ concentration during NLRP3 inflammasome activation using a K^+^ ion fluorescence probe. Spermine treatment significantly restrained K^+^ efflux in macrophages undergoing pyroptosis (Figure 7E). Additionally, we assessed speck formation by ASC, which serves as an indicator of inflammasome assembly. We observed that ASC speck formation induced by LPS plus Nig was inhibited upon spermine treatment (Figure S9C-D). These data suggest that spermine inhibits K^+^ efflux associated with NLRP3 inflammasome activation in macrophages.

**Figure 8:**
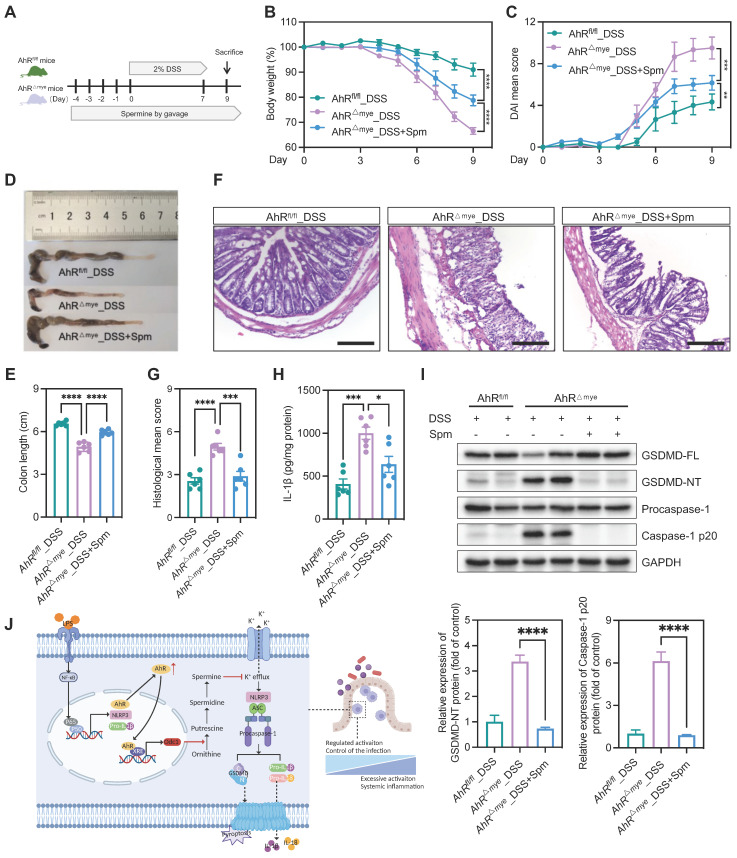
** Spermine confers protection against intestinal inflammation.** (A) Schematic illustration of DSS-induced colitis. Age- and sex-matched AhR^△Mye^ and AhR^fl/fl^ mice were administered 2% DSS in drinking water for 7 days, followed by normal water for 2 days. Mice in spermine treatment groups received daily gavage of Spm for 14 days. (B) Body weight changes were monitored daily and are presented as the percentage of initial body weight. (C) DAI was scored daily for each AhR^△Mye^ and AhR^fl/fl^ mouse based on stool consistency, fecal bleeding, and body weight loss. (D) Representative morphology images of colon specimens from each group. (E) Colon lengths were measured and recorded on day 9. (F) Representative histopathological images of H&E-stained colon sections. Scale bar, 200 μm. (G) Semiquantitative scoring of histopathology. (H) IL-1β expression in colon tissues determined by ELISA. (I) Immunoblot analysis of full-length and cleaved GSDMD and caspase-1 in colon tissue from DSS-treated AhR^△Mye^ and AhR^fl/fl^ mice. n = 6 per group. (J) Proposed working model of AhR confers protection against macrophage pyroptosis and intestinal inflammation. Statistical analysis of the data was performed using one-way ANOVA (E, G, H, I) and two-way ANOVA (B, C) followed by Tukey's multiple comparison tests. Data are shown as the mean ± SEM. *P < 0.05, **P < 0.01, ***P < 0.001, and ****P < 0.0001. AhR, aryl hydrocarbon receptor; DSS, dextran sulfate sodium; DAI, disease activity index; ELISA, enzyme-linked immunosorbent assay; HE, hematoxylin and eosin; Spm, spermine.

We then tested the *in vivo* effect of spermine supplementation in the DSS-induced colitis model. Mice were treated with spermine by daily oral gavage, beginning from 4 days before DSS treatment until the end of the experiments (Figure 8A). Administration of spermine alone had no significant impact on body weight, colon length, or colonic structure (Figure S10A-E). We also assessed the effect of spermine supplementation in this model and found that spermine levels were increased in the colon of mice following spermine oral gavage compared to control vehicle mice (Figure S10F). AhR^fl/fl^ and AhR^△Mye^ mice began to lose body weight on day 4 after starting DSS treatment and AhR^△Mye^ mice showed more body weight loss than their AhR^fl/fl^ littermates (Figure 8B). Remarkably, DSS-induced body weight loss, DAI score, and colon shorting were rescued in AhR^△Mye^ mice treated with spermine (Figure 8B-E). Further, immune cell infiltration and epithelial damage were significantly aggravated in AhR^△Mye^ mice compared with AhR^fl/fl^ mice, and these features were rescued in AhR^△Mye^ mice receiving spermine (Figures 8F-G). DSS-induced pyroptosis was significantly increased in AhR^△Mye^ mice compared with AhR^fl/fl^ mice, and treatment with spermine significantly reduced the expression of caspase-1 p20 and GSDMD-NT, as well as IL-1β production in colon tissue from AhR^△Mye^ mice (Figure 8H-I). Thus, spermine treatment significantly inhibited macrophage pyroptosis and ameliorated DSS-induced colitis in AhR^△Mye^ mice.

## Discussion

In the present study, we identified a critical role for AhR in regulating macrophage pyroptosis. Using macrophage-specific AhR-deficient mice, we found that AhR^△mye^ mice developed severe colitis on DSS treatment, characterized by a significant increase in macrophage pyroptosis and subsequent IL-1β release in colon tissues, compared with AhR^fl/fl^ littermates. Mechanistic studies showed that AhR inhibited macrophage pyroptosis, in part via promoting *Odc1* transcription, to enhance polyamine biosynthesis. The increased polyamine levels, particularly spermine, inhibited NLRP3 inflammasome assembly and subsequent pyroptosis by suppressing K^+^ efflux. Therefore, our findings reveal a potentially novel mechanism whereby the AhR-ODC1-polyamine-pyroptosis axis confers protection against experimental colitis and maintains intestinal immune homeostasis (Figure 8J).

AhR modulates intestinal homeostasis by targeting the development, differentiation, function, and maintenance of several key mucosal immune cell types in the gut 11, 12, 14, 41, 42. While the molecular basis underlying these processes remains unclear, previous studies have shown that administration of the AhR agonist, FICZ, ameliorated colitis severity by downregulating pro-inflammatory cytokines and upregulating IL-22 production in DSS-, 2,6,4-trinitrobenzene sulfonic acid (TNBS)-, and T-cell transfer-induced colitis 34. In contrast, treatment of mice with an AhR antagonist reduced IL-22 production and increased the severity of inflammation in a TNBS-induced mouse colitis model 34. DSS-induced colitis severity was also attenuated by the toxic AhR ligand, 2,3,7,8-tetrachlorodi-benzo-p-dioxin (TCDD), via promoting regulatory T cell (Treg) differentiation and reducing Th17 cell induction through epigenetic regulation 14. It is established that AhR functions are cell type- 43, 44, ligand- 45 and context- 46 dependent. Since* AHR* gene expression is increased, while *CYP1A1* and* CYP1A2* expression showed no difference, in inflamed tissue from patients with UC compared with healthy controls, it is tempting to speculate that AhR upregulation with inadequate activation may be a secondary protective feedback response. Therefore, it is necessary to improve AhR activation to fine-tune the imbalance between pro- and anti- inflammatory factors in patients with IBD. Using genetically modified mouse strains with cell type-specific AhR deletion, we found that mice with AhR deficient in macrophages developed more severe DSS-induced colonic damage. Moreover, dietary supplementation with an AhR pro-ligand, I3C, showed a protective effect in AhR^fl/fl^, but not AhR^△mye^ colitis model mice, highlighting the critical role of macrophage AhR in regulating intestinal inflammation. In particular, AhR-mediated spermine production was shown to confer protection against macrophage pyroptosis and intestinal inflammation, adding a potential new dimension to understanding of AhR-regulated intestinal immune responses.

A constant balance between tissue-resident macrophages and recruited circulating monocytes is critical for maintaining homeostasis in a healthy gut and ensuring protective immunity during infection and autoimmunity 15. ScRNA-seq analysis revealed that the process of circulating monocytes differentiation into tissue resident macrophages is disrupted during colitis, leading to an increased proportion of Ly6c^int^Cx3xr1^int^ macrophages in the LP, which are the main source of IL-1β and IL-6 47. In addition, the proportion of macrophage subsets and the expression of pro-inflammatory cytokines, including IL-1β and IL-6, were significantly elevated in AhR^△mye^ mice compared with those in AhR^fl/fl^ littermates. AhR can negatively regulate LPS-induced inflammatory responses through its interaction with the NF-κB pathway via Stat1, to inhibit IL-6 expression 18 and, via plasminogen activator inhibitor-2 (Pai-2), to inhibit IL-1β secretion 19. AhR activation by TCDD negatively regulates NLRP3 inflammasome activity by inhibiting *NLRP3* transcription 48. We found that AhR^-/-^ macrophages secreted much larger amounts of IL-1β on NLRP3 inflammasome activation, likely due to enhanced IL-1β processing, rather than synthesis. Since IL-1β is the secreted effector protein of pyroptosis, we found that AhR functions as a suppressor of macrophage pyroptosis via targeting polyamine biosynthesis. Pyroptosis-associated proteins were upregulated in colonic tissues from AhR^△mye^ mice compared with those in AhR^fl/fl^ littermates, which may have contributed to the development of intestinal mucosal inflammation in AhR^△mye^ mice. Over-secretion of IL-1β by AhR-deficient macrophages suggests that AhR may function as a suppressor of macrophage pyroptosis, and even as a physiological immune suppressor. There is an apparent discrepancy between our observation of increased macrophage numbers in the colon of AhR^△Mye^ mice following DSS challenge and the protective role of AhR against macrophage pyroptosis. The overall inflammatory environment created by DSS challenge and pyroptosis-induced cytokine release can attract immune cells, including macrophages, to the inflammation site. This amplification of the immune response can result in an increase in macrophage numbers, even if some macrophages undergo pyroptosis. Compensatory mechanisms may also enhance macrophage recruitment, proliferation, or survival to counteract the loss due to pyroptosis. Therefore, we also believe that AhR deficiency may affect the migration or recruitment of macrophages, possibly due to direct or indirect induction by macrophage pyroptosis.

Pyroptosis has been proposed to play an important role in the pathogenesis of colitis, although the results remain controversial. Some studies showed that NLRP3^-/-^ deficiency resulted in decreased intestinal inflammation 16, 49, while other investigations have suggested that abnormal activation of the NLRP3 inflammasome leads to release of pro-inflammatory cytokines, such as IL-1β and IL-18, which disrupt the intestinal barrier 50, 51. We found that the NLRP3 inflammasome was overactivated in mice in the DSS-treated group, and that spermine treatment suppressed expression levels of IL-1β and the pyroptosis executive proteins, caspase-1 p20 and GSDMD-NT. It is likely that pyroptosis activation in IBD occurs in the epithelium and plays a protective role, as it would be expected to maintain homeostasis through regulation of commensal microbiota and eradication of harmful bacterial; however, once the epithelial barrier is disrupted, gut microbiota will infiltrate into the LP and recruit immune cells; in this case, pyroptosis would be predicted to provoke a cytokine storm, which would damage the mucosa. Interestingly, a recent study reported that full-length GSDMD in epithelial cells plays a non-pyroptotic role in promoting the release of IL-1β-containing small extracellular vesicles 52. Rana *et al.* also reported that GSDMB facilitates epithelial restitution and repair, dependent on PDGF-A-mediated FAK phosphorylation, but not pyroptosis 53. The role of pyroptosis in IBD is emerging as the focus of increasing attention, and a clear understanding of the mechanism underlying pyroptosis will guide the development of future effective therapeutics for IBD.

*Odc1* mRNA expression levels were markedly reduced in AhR^-/-^ BMDMs, similar to a previous finding in normal human fibroblast (WI-38) cell line 54. Additionally, we noticed that AHR is not induced in inactive disease, whereas ODC1 is. This suggests that ODC1 might also be AHR-independently regulated. Here are several alternative mechanisms to consider. First, microbiota-derived signals, such as LPS, can stimulate ODC activity through toll-like receptor signaling pathways independently of AhR activation 55. Second, metabolic changes associated with inflammation, such as increased glycolysis or polyamine demand, can stimulate ODC1 expression to support cellular functions 56. Third, inflammatory cytokines, such as TNF-α and IL-1β, are often elevated in inflammatory conditions and can activate downstream signaling pathways, including NF-κB, which in turn can enhance ODC1 transcription 57. ODC1 is the first and rate-limiting enzyme in polyamine metabolism. Polyamines, including putrescine, spermidine, and spermine, are abundant within the gastrointestinal tract and are essential for cell viability, proliferation, function, and differentiation 58. Natural polyamines are reported to have potential as an adjunctive treatment for colitis in mouse models. Nishiguchi *et al.* developed oral anti-inflammatory polyamine-based nanomedicines that exhibited great potential as biocompatible ROS scavenger drugs that suppressed inflammatory responses in a UC colitis mouse model 59. Microbial-derived putrescine increased the abundance of anti-inflammatory macrophages in the colon, and ameliorated DSS-induced colitis 60. Exogenous supplementation with putrescine or ornithine enhanced ILC3 production of IL-22, and protected mice against *Citrobacter rodentium* infection 61. Spermidine supplementation reversed the DSS-induced colitis phenotype associated with increased expression of α-defensins and a shift in microbiome 62, M1/M2 macrophage polarization 63, and enhanced hypusination of intestinal epithelial cells 64. Here we show that spermine supplementation can inhibit macrophage pyroptosis *in vitro* and ameliorate colon damage *in vivo*, which might be related to potassium channel regulation, suggesting its potential utility against IBD.

In summary, our study highlights a potentially novel regulatory pathway, involving the AhR-ODC1-polyamine-pyroptosis axis, in IBD. The abundance of natural AhR ligands and polyamines found in the environment provide a positive outlook for future therapeutic agents targeting AhR or polyamines in macrophages, as immunomodulators through their inhibitory effects on macrophage pyroptosis.

## Methods

### Mice

All mice used in this study were on a C57BL/6 background. AhR-null mice (AhR KO, B6.129-Ahr^tm1Bra^/J) and AhR^fl/fl^ (B6.129(FVB)-Ahr^tm3.1Bra^/J) mice were purchased from the Jackson Laboratory (Bar Harbor, ME, USA). Lyz2-Cre mice [B6.129P2-Lyz2^tm1(cre)Ifo^/J] were obtained from Shanghai Model Organisms (Shanghai, China). AhR^fl/fl^ mice bearing two loxP sites flanking the second exon of the* AhR* gene were cross-bred with Lyz2-Cre mice to specifically knockout AhR in the myeloid cell lineage, including macrophages (termed AhR^△Mye^ mice). Mouse genotyping primers are listed in Supplementary Table S1. C57BL/6 mice were obtained from the SLAC Laboratory Animal Co. (Shanghai, China). All animal experiments complied with relevant laws and institutional guidelines, as overseen by the Committee on Animal Research and Ethics of Fudan University (Shanghai, China). All animals were housed, bred, and maintained under specific pathogen-free conditions.

### DSS-induced mouse colitis model

A DSS-induced mouse colitis model was established as described previously, with slight modifications 33. Briefly, age- and sex-matched AhR^+/+^, AhR^-/-^, AhR^fl/fl^, and AhR^△Mye^ mice were administered DSS (molecular weight, 36-50 kDa; MP Biomedicals, Santa Ana, CA, USA) in drinking water for 7 days, followed by regular drinking water for 2 days. Our study examined male and female animals, and similar findings are reported for both sexes. Control mice were provided with normal drinking water. For pharmacological treatment, I3C (Sigma, Milpitas, CA, USA) or spermine (Sigma, Milpitas, CA, USA) were dissolved in DMSO and further diluted with ddH_2_O to generate working concentration solutions. Animals were treated with or without I3C (1 mg per mouse in 100 μL ddH_2_O) or spermine (50 mg/kg body weight in 100 μL ddH_2_O) by daily oral gavage, beginning from 4 days before DSS treatment until the end of the experiments. Mice in the control group received an equal volume gavage of vehicle. Mice were sacrificed in an automated CO_2_ delivery system, and their colons collected immediately for colon length measurement, protein extraction, and histology analysis.

### Determination of disease activity index (DAI)

DAI was scored daily for each mouse, based on body weight loss, stool consistency, and gross bleeding. Scores were evaluated, as follows: body weight loss, depicted as the percentage of initial body weight: 0 (no loss), 1 (1% to 5% loss), 2 (6% to 10% loss), 3 (11% to 15% loss), 4 (> 15% loss); stool consistency: 0 (normal), 2 (pasty and semi-formed stools, did not adhere to the anus), 4 (liquid stools that did adhere to the anus); bleeding: 0 (no blood), 1 (positive hemoccult), 4 (gross bleeding).

### Histologic analysis

Colon tissues were fixed in 4% neutral-buffered formalin. Paraffin-embedded colon sections (5 μm) were stained with hematoxylin and eosin according to manufacturer's protocols (Wuhan Servicebio Technology, Wuhan, China). Histological score was determined by combining scores for inflammatory cell infiltration (0 to 3) and tissue damage (0 to 3). The presence of inflammatory cells in the intestinal LP were evaluated as follows: 0, occasional inflammatory cells in the LP; 1, increased numbers of inflammatory cells in the LP; 2, confluence of inflammatory cells extending into the submucosa; 3, transmural extension of the infiltrate. Tissue damage was scored as follows: 0, no mucosal damage; 1, lymphoepithelial lesions; 2, surface mucosal erosion or focal ulceration; 3, extensive mucosal damage and extension into deeper structures of the bowel wall.

### LPS-induced inflammation

Mice were injected intraperitoneally with 200 μL LPS (20 mg/kg,* Escherichia coli* 055: B5, Sigma, Milpitas, CA, USA) in sterile PBS. To determine survival rate, mice were constantly monitored every 6 h for a total of 60 h. In a separate experiment, mice were sacrificed 16 h after LPS (10 mg/kg) injection. Serum samples were collected for enzyme-linked immunosorbent assay (ELISA) of IL-1β and IL-6.

### Cell culture, stimulation, and transfection

Mouse primary BMDMs were generated as previously described 65. Briefly, bone marrow was extracted from femurs and tibias. Then, after lysis of erythrocytes, plated in complete medium consisting of Dulbecco's modified Eagle's medium (DMEM; Gibco, Carlsbad, CA, USA) containing 10% fetal bovine serum (FBS; Gibco, Carlsbad, CA, USA) and 10 ng/mL macrophage colony-stimulating factor-1 (M-CSF; R&D System, Minneapolis, MN, USA). Adherent macrophages were obtained after 7 days of culture. No differences in differentiation were observed between WT and AhR^-/-^ BMDMs. RAW264.7 cells were purchased from the Cell Bank, Shanghai Institute for Biological Science, Chinese Academy of Science, and cultured in DMEM medium supplemented with 10% FBS. All cells were maintained in humidified cell incubators in 5% CO_2_ at 37 °C.

Transient transfection of siRNAs (GenePharma, Shanghai, China) was performed using Lipofectamine RNAiMAX reagent (Invitrogen, Carlsbad, CA, USA) and transfection of plasmids with Lipofectamine 2000 reagent (Invitrogen, Carlsbad, CA, USA), according to the manufacturer's instructions. siRNA sequences are listed in Supplementary Table S2.

To activate the canonical NLRP3 inflammasome, BMDMs were primed with 200 ng/mL LPS (Sigma, Milpitas, CA, USA) for 4 h, followed by 10 μM Nig (Merck, Darmstadt, Germany) or 5 mM ATP (Sigma, Milpitas, CA, USA) for 30-60 min. For pharmacological treatment, VX-765 (50 μM; Merck, Darmstadt, Germany) was added 30 min prior to LPS treatment. Spermine (50 μM; Sigma, Milpitas, CA, USA) was added together with LPS for 4 h prior to Nig stimulation.

### Microscopy imaging of cell death

To examine cell death morphology, cells were treated as indicated, then propidium iodide (PI; BD, Franklin Lake, NJ, USA) added to cell cultures at a final concentration of 1 μg/mL to monitor cell membrane integrity. Cells were washed with PBS and static field images of pyroptotic cells captured using a microscope (Leica, Weztlar, Germany).

### Cytotoxicity assays

BMDMs were seeded at 3 × 10^4^ cells per well in 96-well plates. After stimulation, cell supernatants were collected and centrifuged at 500 × g for 5 min to remove cellular debris. Cell death was determined by LDH release assay using a CytoTox 96 Non-Radioactive Cytotoxicity Assay kit (Promega, Madison, WI, USA), according to the manufacturer's instructions. Absorbance was read at 492 nm using a plate reader. Data were normalized relative to positive lysis wells (set as 100% lysis) treated with 0.1% Triton X-100.

### Enzyme-linked immunosorbent assay (ELISA)

Mouse IL-1β, IL-6, and TNFα protein levels were measured using DuoSet ELISA kits (Invitrogen, Carlsbad, CA, USA), according to the manufacturer's instructions. Absorbance was read at 450 nm using a plate reader.

### Plasmid construction

pEGFP-C3-AhR was generated by inserting *AhR* cDNA fragments, generated from mouse cDNA by PCR amplification, into the HindIII and SacII restriction sites in the multiple cloning site of pEGFP-C3. A 2.0-kb fragment upstream of the *Odc1* transcription start site was generated by PCR using mouse genomic DNA as template and cloned into the pGL3-Basic Vector using a ClonExpression® II One Step Cloning Kit (Vazyme, Nanjing, China), according to manufacturer's instructions. pcDNA3.1-flag-ODC1 was generated by cloning* Odc1* cDNA fragments into the pcDNA3.1-flag vector using a ClonExpression® II One Step Cloning Kit (Vazyme, Nanjing, China), according to the manufacturer's instructions. Sense and anti-sense primers used are listed in Supplementary Table S3.

### Luciferase reporter assay

Luciferase activity was measured using the Dual-luciferase Reporter Assay system (Promega, Madison, WI, USA), according to the manufacturer's instructions. Briefly, HEK293T cells (2 × 10^4^ cells/well) were plated in 24-well plates, and co-transfected with a mixture of the luciferase reporter plasmid, pRT-TK-Renilla, and the indicated expression plasmids using Lipofectamine 2000 (Invitrogen, Carlsbad, CA, USA). Post-transfection (48 h), luciferase activities were measured using a Dual luciferase Reporter Assay System. Data were normalized to transfection efficiency by dividing firefly luciferase activity by Renilla luciferase activity.

### Visualization of ASC speck formation

BMDMs were seeded onto glass coverslips in 6-well plates. Cells were incubated with LPS (200 ng/mL) for 4 h followed by Nig (10 μM) for 45 min, then washed with PBS, fixed in 4% paraformaldehyde, and permeabilized in 0.3% Triton X-100. After three washes, cells were blocked with 5% BSA in PBS. Anti-ASC antibody (Adipogen, San Diego, CA, USA) was added at 5 μg/mL and incubated at 4 ℃ overnight. After washing, cells were incubated with alexa fluor 647-conjugated secondary antibody (1:500; Beyotime, Shanghai, China) for 1 h at room temperature. Finally, cells were stained with DAPI (Beyotime, Shanghai, China) and sealed for visual analyses. Images were captured by using a laser confocal microscope (Leica, Weztlar, Germany).

### Immunofluorescence staining and confocal analysis

Mouse Colon tissues were fixed in 4% neutral-buffered formalin. Paraffin-embedded colon sections (5 μm) were deparaffinized, rehydrated, and fixed. After antigen retrieval, tissues were blocked with 3% BSA, then incubated with anti-mouse F4/80 (Servicebio, Wuhan, China) and anti-mouse IL-1β (Servicebio, Wuhan, China) at 4 °C overnight. After washing, tissue specimens were incubated with alexa fluor 488- and alexa fluor 647-conjugated secondary antibodies (Servicebio, Wuhan, China) for 1 h at room temperature. Nuclei were stained with DAPI. Images were captured using a laser confocal microscope (Leica, Weztlar, Germany).

### Quantitative reverse transcription PCR (RT-qPCR)

Total RNA was extracted from cells using TRIzol Reagent (Invitrogen, Carlsbad, CA, USA). A PrimeScript II 1st Strand cDNA Synthesis Kit (Takara, Japan) was used to synthesize first-strand cDNA. RT-qPCR was performed using SYBR® Premix Ex Taq™ II (Takara, Japan) on a Roche 480 Real Time PCR System. mRNA levels of target genes were normalized to those of β-actin. Differences in expression levels (fold-change) were calculated using the 2^-ΔΔCT^ method. PCR primers used are listed in Supplementary Table S4.

### Protein preparation and immunoblotting

Cell and tissue protein lysates were extracted using radio immunoprecipitation assay lysis buffer (Thermo Fisher Scientific, Waltham, MA, USA) containing protease and phosphatase inhibitors (Thermo Fisher Scientific, Waltham, MA, USA). Proteins in cell culture supernatants were precipitated using methanol-chloroform, as described previously 66. Protein concentrations were determined using BCA Reagent (Takara, Japan). Protein aliquots (30 μg) were separated by sodium dodecyl sulfate polyacrylamide gel electrophoresis, transferred to PVDF membranes, and probed with antibodies directed against β-tubulin (Abcam, Cambridge, UK), GSDMD (L60) (Cell Signaling Technology, Boston, MA, USA), Caspase-1 p20 (Casper-1) (Adipogen, San Diego, CA, USA), IL-1β (Cell Signaling Technology, Boston, MA, USA), NLRP3 (Cryo-2) (Adipogen, San Diego, CA, USA), ASC (AL177) (Adipogen, San Diego, CA, USA), AhR (Enzo Lifesciences, Farmington, NYC, USA), ODC1 (Proteintech, Wuhan, China), or GAPDH (Proteintech, Wuhan, China). Bands were visualized using chemiluminescent HRP substrate (Thermo Fisher Scientific, Waltham, MA, USA) and a Molecular Imager® ChemiDoc^TM^ XRS+ Imaging System (Bio-Rad, Hercules, CA, USA).

### Chromatin immunoprecipitation (ChIP)

ChIP assays were performed using a Simple ChIP® Plus Enzymatic Chromatin IP Kit (Cell Signaling Technology, Boston, MA, USA), according to the manufacturer's instructions. Briefly, cells were fixed with 1.5% formaldehyde for 20 min and quenched with glycine for 5 min. Chromatin was fragmented by micrococcal nuclease digestion and sonication and immunoprecipitated with an anti-AhR (Enzo Lifesciences, Farmington, NYC, USA) or a control IgG antibody, overnight at 4 °C. Antibody-chromatin complexes were then pulled down using ChIP-Grade Protein A/G Magnetic Beads and eluted with Elution buffer. After incubation at 65 °C for crosslink reversal, samples were digested with RNase A and proteinase K. Immunoprecipitated DNA was collected using DNA clean-up columns. DNA was quantified by qPCR using SYBR® Premix Ex Taq™ II (Takara, Japan), according to the manufacturer's instructions. The PCR primers used are listed in Supplementary Table S5.

### RNA sequencing and data analysis

Total RNA was extracted from WT and AhR^-/-^ BMDMs for RNA-seq analysis. Qualified RNA samples were used for library construction, and then sequenced (PE150) on an Illumina Novaseq™ 6000 (LC-Bio Technology CO., Ltd., Hangzhou, China), to generate 2 × 150 bp long reads, following the vendor's recommended protocol. After removing adaptors and low-quality bases from raw data, high-quality sequences (clean data) were mapped to the *Mus musculus* GRCm38 reference genome*.* Differentially expressed genes were selected as those with false discovery rate < 0.5, fold-change ≥ 1.5 or ≤ 0.5, and p value < 0.05, using the R package, edgeR. RNA-seq data have been deposited in the SRA database under accession number PRJNA952314.

### ScRNA-seq library preparation and analysis

AhR-null and WT mice were administered 2% DSS in drinking water for 7 days, followed by normal drinking water to induced acute colitis. On day 8, colon tissues from three mice of the same group were randomly mixed as one sample, then, colons were digested and single cell suspensions obtained using 70-μm filters (BD, Franklin Lake, NJ, USA). After incubation with red blood cell lysis buffer, CD45^+^ immune cells were isolated by positive selection with anti-mouse CD45 magnetic beads (Miltenyi, Germany), according to the manufacturer's instructions. Finally, cells were resuspended in complete RPMI-1640 medium, counted for viability, and then immediately subjected to library preparation. ScRNA-Seq libraries were prepared using 10x Single Cell Genomics technology (10x Genomics, San Diego, CA, USA), according to the manufacturer's instructions. ScRNA-seq fastq was applied, with default parameters, to filter adaptor sequences and remove low-quality reads. Graph-based clustering was performed for cell clustering according to their gene expression profiles. Cells were visualized using a 2-dimensional UMAP algorithm with the RunUMAP function in Seurat. The FindAllMarkers function in Seurat was applied to identify marker genes for each cluster. Sequencing and bioinformatics analysis were conducted by OE Biotech Co., Ltd. (Shanghai, China). ScRNA-seq data have been deposited in the GEO database under accession number GSE242968.

### 16S rDNA sequencing

Microbial DNA was extracted from fecal samples from AhR^△mye^ mice and AhR^fl/fl^ littermates using a DNeasy PowerSoil kit (Qiagen, Hilden, Germany), following the manufacturer's instructions. DNA concentration and integrity were verified by NanoDrop 2000 analysis and agarose gel electrophoresis, respectively. Genomic DNA was used as template for PCR amplification with barcoded primers and Tks Gflex DNA Polymerase (Takara, Japan). After amplification and purification, PCR products were quantified using a Qubit dsDNA assay kit (Life Technologies, Carlsbad, CA, USA). Sequencing was performed on an Illumina NovaSeq 6000 (Illumina lnc., San Diego, CA; OE Biotech Company, Shanghai, China). 16S rDNA-seq data have been deposited in the SRA database under accession number PRJNA1002140.

### Intracellular polyamine analysis

For harvest, BMDMs (1 × 10^7^) were rinsed with 500 μL pre-cooled methanol:water (2:1, v/v) and subjected to three freeze-thaw cycles before sonication in an ice bath for 15 mins. Following centrifugation, supernatants were evaporated to dryness and re-dissolved in 50 μL of acetonitrile-water (1:4, v/v). Next, aliquots (10 μL) were vortex-mixed with 10 μL of NEM solution (20 mM) in phosphate buffer (0.1 M, pH 7.0) containing ascorbic acid (10 mM) and EDTA (10 mM) for 1 min. tBBT solution (10 μL, 0.23 M) was added, followed by 87 μL borate buffer (0.2 M, pH 8.8) containing TCEP (20 mM) and ascorbic acid (5 mM). After vortex-mixing, 33 μL 5-AIQC solution was added and samples incubated at 55 °C for 10 min. Mixtures were cooled and 2 μL formic acid added, followed by centrifugation at 13,000 ×g for 10 min at 4 °C, and supernatants filtered through 0.22 µm filters before UPLC-MS/MS analysis. Metabolite analysis was carried out using an Agilent 1290 UPLC coupled to an Agilent 6470 triple quadrupole mass spectrometer equipped with an electrospray ionization source (Agilent Technologies, Palo Alto, CA, USA). Chromatographic separation of polyamines was performed using a UPLC column (Agilent ZORBAX RRHD Eclipse XDB C18 column, 2.1 × 100 mm, 1.8 μm particles) with a solvent gradient of buffer A (water) and buffer B (methanol containing 0.1% (v/v) formic acid); flow rate, 0.5 mL/min. An optimized gradient elution scheme was employed as follows: 1% B (0-2 min), 1%-3.8% B (2-4 min), 3.8%-14% B (4-7.3 min), 14%-22% B (7.3-10.7 min), 22%-24% B (10.7-14.7 min), 24%-30% B (14.7-16 min), 30%-60% B (16-16.3 min), 60%-70% B (16.3-17.3 min), 70%-95% B (17.3-17.31 min), and 95% B (17.31-20 min). Electrospray ionization was performed in positive ion mode. Multiple reaction monitoring was applied for quantification of screening fragment ions. Peak determination and peak area integration were performed using MassHunter Workstation software (Agilent, Version B.08.00). Standard curves were constructed by least-squares linear regression analysis, using the peak area ratio of derivatized individual standards against the nominal concentration of the calibrator. Quantification was performed identically for all samples. Detection of polyamines was conducted by Shanghai Metabolome Institute (SMI)-Wuhan.

### Intracellular potassium (K^+^) analysis

BMDMs were seeded in 48-well plates at 5 × 10^4^ cells per well. After stimulation, cells were probed with IPG-4 AM (Ion Biosciences, San Marcos, TX, USA), a yellow-green fluorescent, intracellular K^+^ indicator, in a cell culture incubator at 37 °C for 90 min, according to the manufacturer's instructions. After staining, cells were washed and analyzed by flow cytometry (BD, Franklin Lake, NJ, USA). Mean fluorescence intensity was calculated using FlowJo software (BD, Version 10.8.1).

### Statistical Analyses

All statistical analyses were performed using GraphPad Prism 9.0 software. Before comparisons between groups, data were tested for Gaussian distribution and homogeneity variance. For data in Gaussian distribution and with homogeneity variance, parametric test was used to analyze, such as independent t test, one or two-way ANOVA, etc. For data in non-Gaussian distribution, non-parametric test was used to analyze, such as the Mann-Whitney test, Kruskal-Wallis test, Spearman correlation, etc. For data in Gaussian distribution and without homogeneity variance, Welch's correction was used. *P* < 0.05 was considered statistically significant and data are presented as mean ± SEM.

### Ethics Committee Approval

All animal experiments complied with all relevant ethical regulations for animal testing and research and were in accordance with protocols approved by the Committee on Animal Research and Ethics of Fudan University.

### Data and materials availability

The raw data from the scRNA-seq, RNA-seq and 16s rDNA-seq have been deposited in the GEO or SRA database under accession numbers GSE242968, PRJNA952314, and PRJNA1002140, respectively. All data needed to evaluate the conclusions in the paper are present in the paper and the Supplementary Materials. Additional data are available from authors upon request.

## Supplementary Material

Supplementary figures and tables.

## Abbreviations

AhR: aryl hydrocarbon receptor
ARNT: aryl hydrocarbon receptor nuclear translocator
ASC: apoptosis associated speck like protein containing a CARD
ATP: adenosine Triphosphate
BMDMs: bone marrow derived macrophages
CD: Crohn's disease
ChIP: chromatin immunoprecipitation
DAI: disease activity index
DSS: dextran sulfate sodium
DFMO: difluoromethylornithine
ELISA: enzyme-linked immunosorbent assay
FICZ: 6-Formylindolo[3,2-b] carbazole
GSDMD: gasdermin D
IBD: inflammatory bowel disease
IL-1-RN: IL-1 antagonist receptor
IL-18RAP: IL18 receptor accessory protein
IL-18R1: IL18 receptor 1
IP: indeno[1,2,3-cd]pyrene
I3C: indole-3-carbinol
ILC: innate lymphoid cells
IEL: intraepithelial lymphocytes
LPS: lipopolysaccharide
LC-MS: liquid chromatograph mass spectrometer
LP: intestinal lamina propria
LDH: lactate dehydrogenase
M-CSF: macrophage colony-stimulating factor-1
Nig: nigericin
NLRP3: NOD-like receptor protein 3
Odc1: ornithine decarboxylase 1
PI: propidium iodide
PRRs: pattern recognition receptors
ROS: reactive oxygen species
scRNA-seq: single-cell RNA sequencing
Spd: spermidine
Spm: spermine
TNBS: 2,6,4-trinitrobenzene sulfonic acid
TCDD: 2,3,7,8-tetrachlorodibenzo-p-dioxin
UC: ulcerative colitis
UMAP: uniform manifold approximation and projection
WT: wild type
XREs: xenobiotic response elements
