# Supplementary material for: Aryl hydrocarbon receptor confers protection against macrophage pyroptosis and intestinal inflammation through regulating polyamine biosynthesis
Source: Theranostics. 2024 Jul 8;14(11):4218–39. doi: 10.7150/thno.95749 (PMC11303072; doi:10.7150/thno.95749)
Supplement: Supplementary file 1 — Supplementary figures and tables. [file thnov14p4218s1.pdf]

Supplement figures

Fig.S1

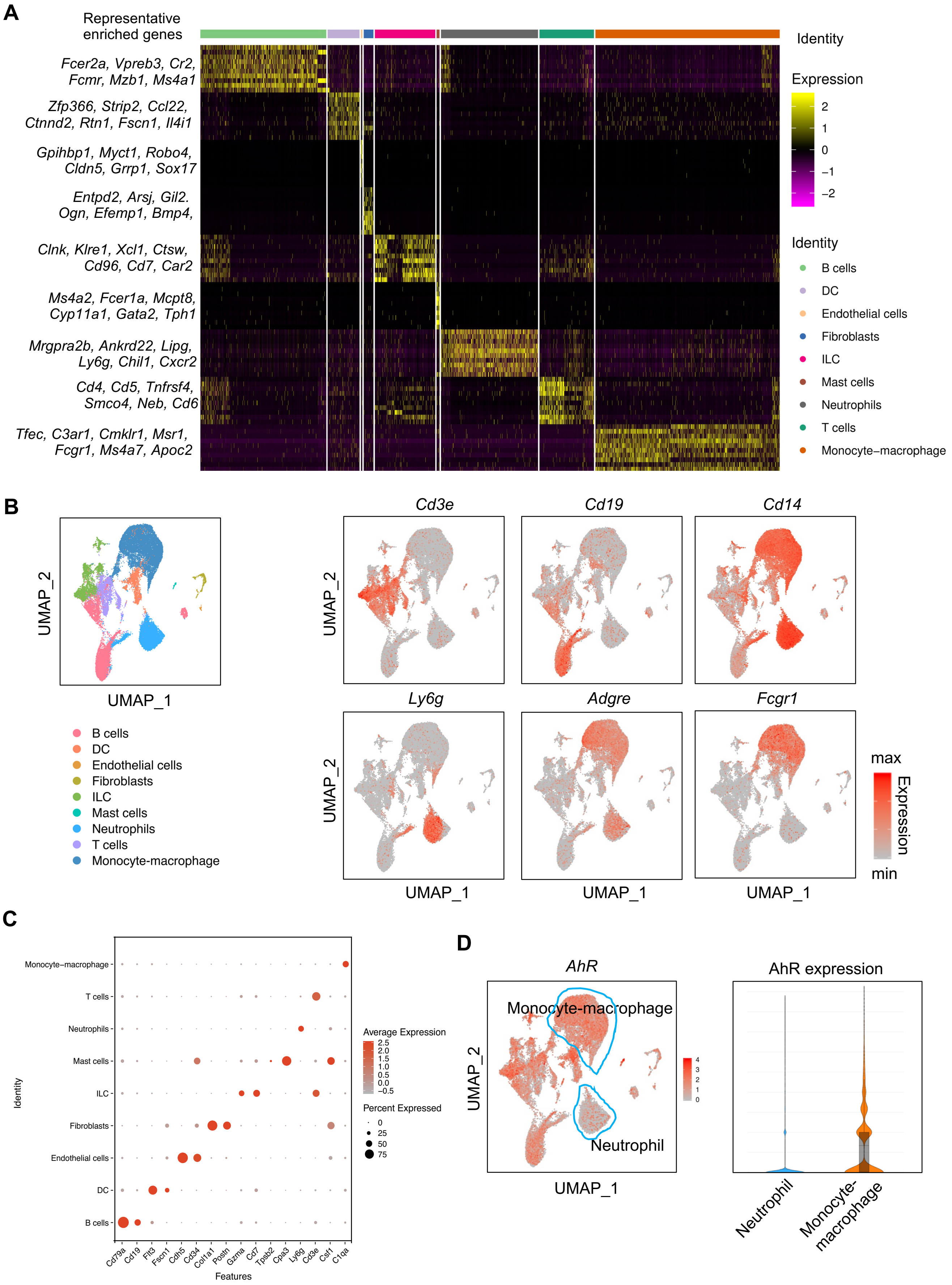

Figure S1. Single-cell RNA sequencing unveils major intestinal lamina propria CD45^{+} immune cell populations in AhR$^{+/+}$ littermates and AhR$^{-/-}$ mice. (A) Cells were divided into nine clusters, and representative genes in each cluster are shown. (B) Expression of the linage marker genes, *Cd3e*, *Cd19*, *Cd14*, *Ly6g*, *Adgre1*, and *Fcgr1* shown in the UMAP plots. Color intensity corresponds to the relative expression level of specific genes. (C) Dot-plot showing unique marker genes for all cell types. (D) AhR expression in the UMAP plots. Color intensity corresponds to the relative expression of AhR. Violin plot comparing AhR expression between neutrophils and monocyte-macrophage lineage. AhR, aryl hydrocarbon receptor; UMAP, uniform manifold approximation and projection.

Fig.S2

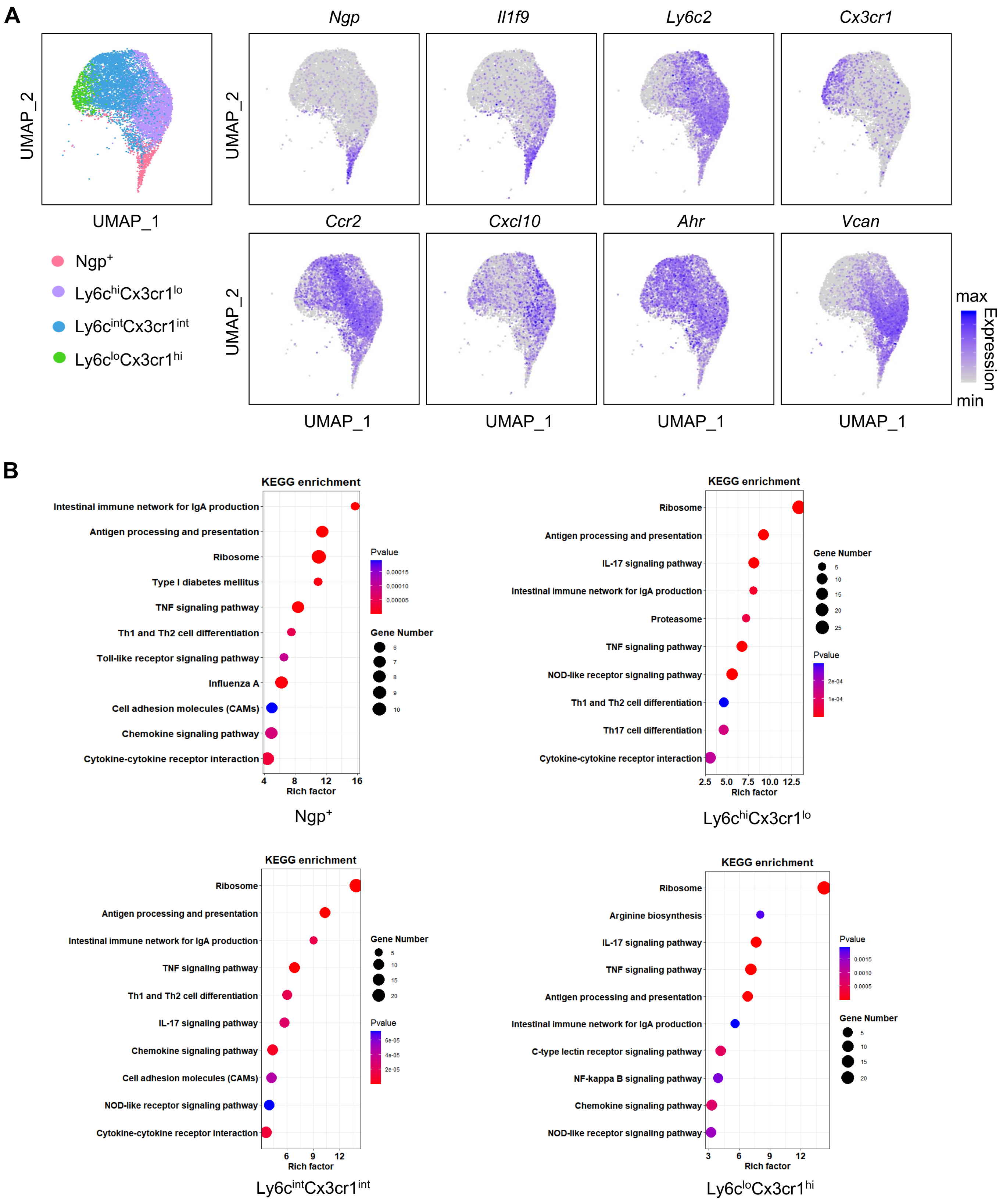

Figure S2. Distinct transcriptional signatures of intestinal monocyte-macrophage lineage within each cluster determined by high-through single-cell RNA sequencing analysis. (A) UMAP and graph visualization defined four clusters among monocyte-macrophage lineage cells. Marker genes for sub-clustering shown in the UMAP plots. Color intensity corresponds to relative gene expression. (B) KEGG analysis to identify pathways enriched in intestinal monocyte-macrophage lineage sub-cluster from AhR$^{-/-}$ and AhR$^{+/+}$ mice. AhR, aryl hydrocarbon receptor; KEGG, Kyoto Encyclopedia of Genes and Genomes; UMAP, uniform manifold approximation and projection.

Fig.S3

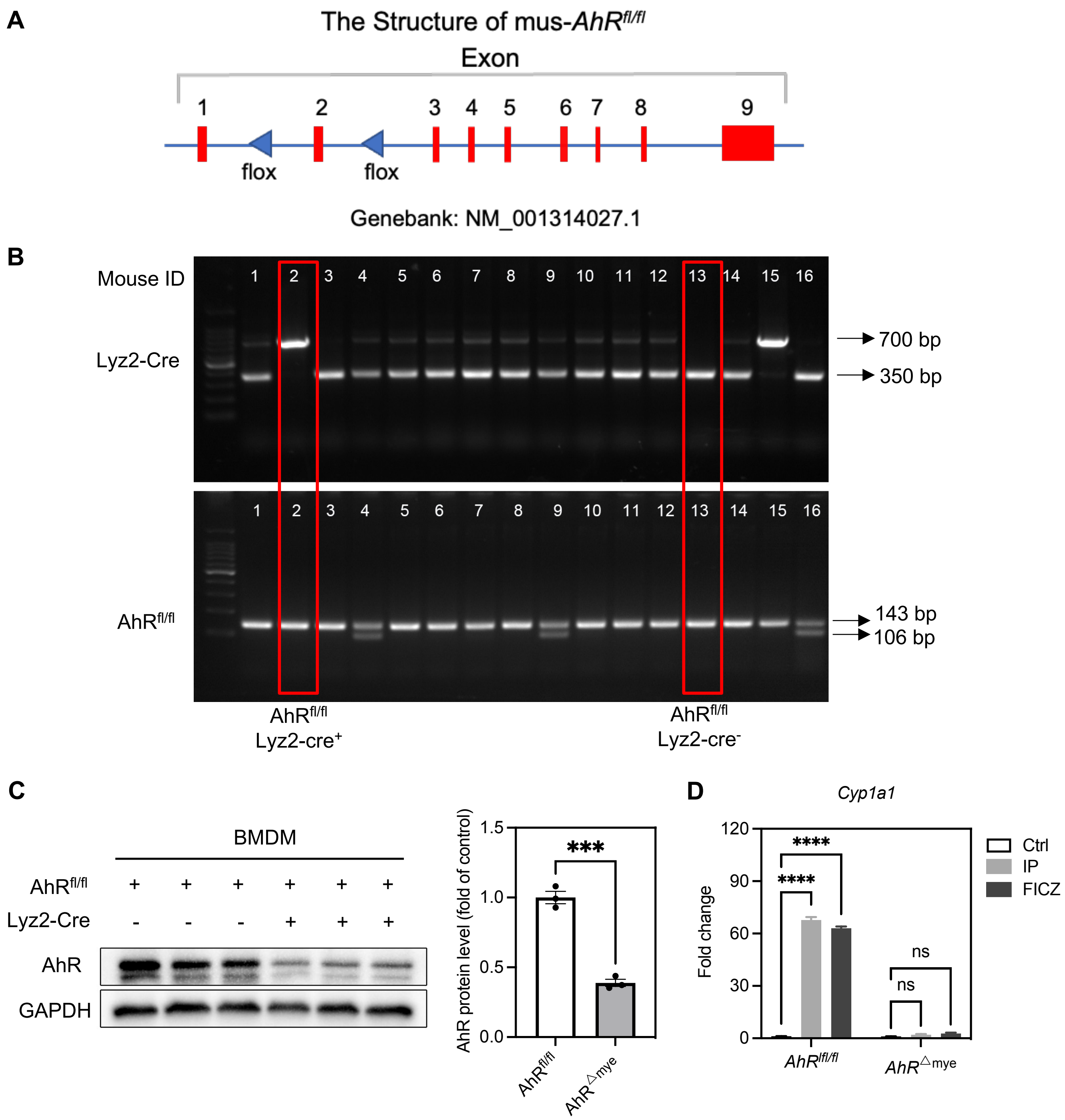

Figure S3. AhR knockout efficiency in macrophages. (A) Schematic diagram of the targeting strategy for AhR depletion in the myeloid compartment using the Cre-LoxP system. (B) PCR results showing the genotype of myeloid-specific AhR KO mice. (C) AhR protein levels were determined in BMDMs from $AhR^{fl/fl}$ and $AhR^{\Delta Mye}$ mice by western blot analysis. (D) BMDMs were treated with IP (500 nM) or FICZ (100 nM) for 24 h and *Cyp1a1* mRNA expression levels quantified by RT-qPCR. Statistical analysis of the data was performed using two-tailed unpaired *t* test (C), and two-way ANOVA (D) followed by Tukey's multiple comparison tests. Data are shown as the mean ± SEM. ns, not significant; $***P < 0.001$, $****P < 0.0001$. AhR, aryl hydrocarbon receptor; BMDMs, bone marrow derived macrophages; FICZ, 6-Formylindolo[3,2-b] carbazole; IP, indeno[1,2,3-cd]pyrene; PCR, Polymerase Chain Reaction.

Fig.S4

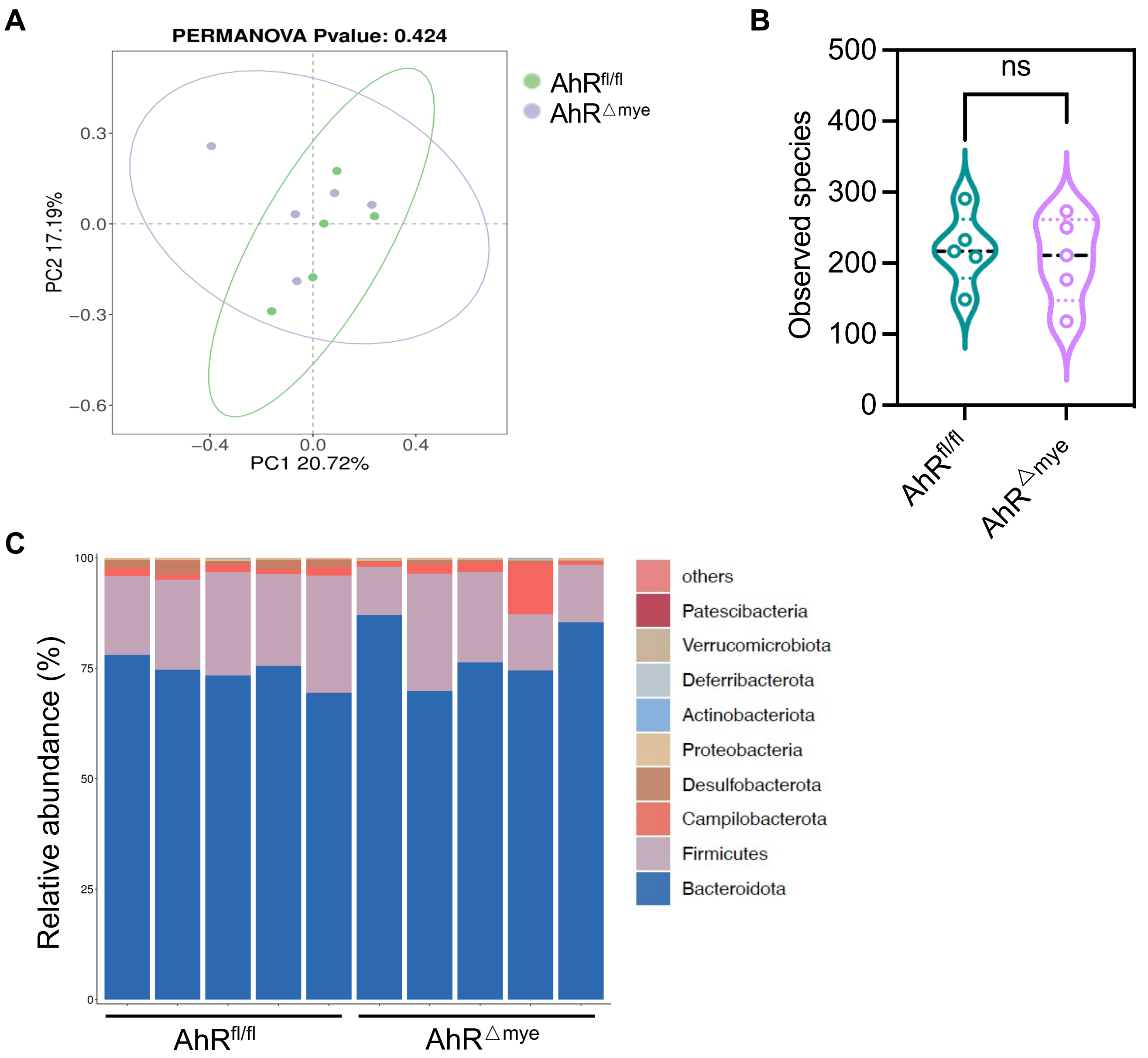

Figure S4. Luminal microbiota composition was comparable between AhR$^{\Delta Mye}$ mice and AhR$^{fl/fl}$ littermates at 8 weeks of age. Fecal samples were collected from AhR$^{\Delta Mye}$ and AhR$^{fl/fl}$ littermates (n = 5) at 8 weeks of age and analyzed by 16s rDNA sequencing. (A) Principal coordinate analysis plot; *P* was determined by permutational multivariate analysis of variance. (B) Alpha diversity, evaluated by the number of observed species. (C) Gut bacterial community composition at the phylum level, expressed as a ratio of the total community. Statistical analysis of the data was performed using two-tailed unpaired *t* test (B). Data are shown as the mean ± SEM. ns, not significant. AhR, aryl hydrocarbon receptor.

Fig.S5

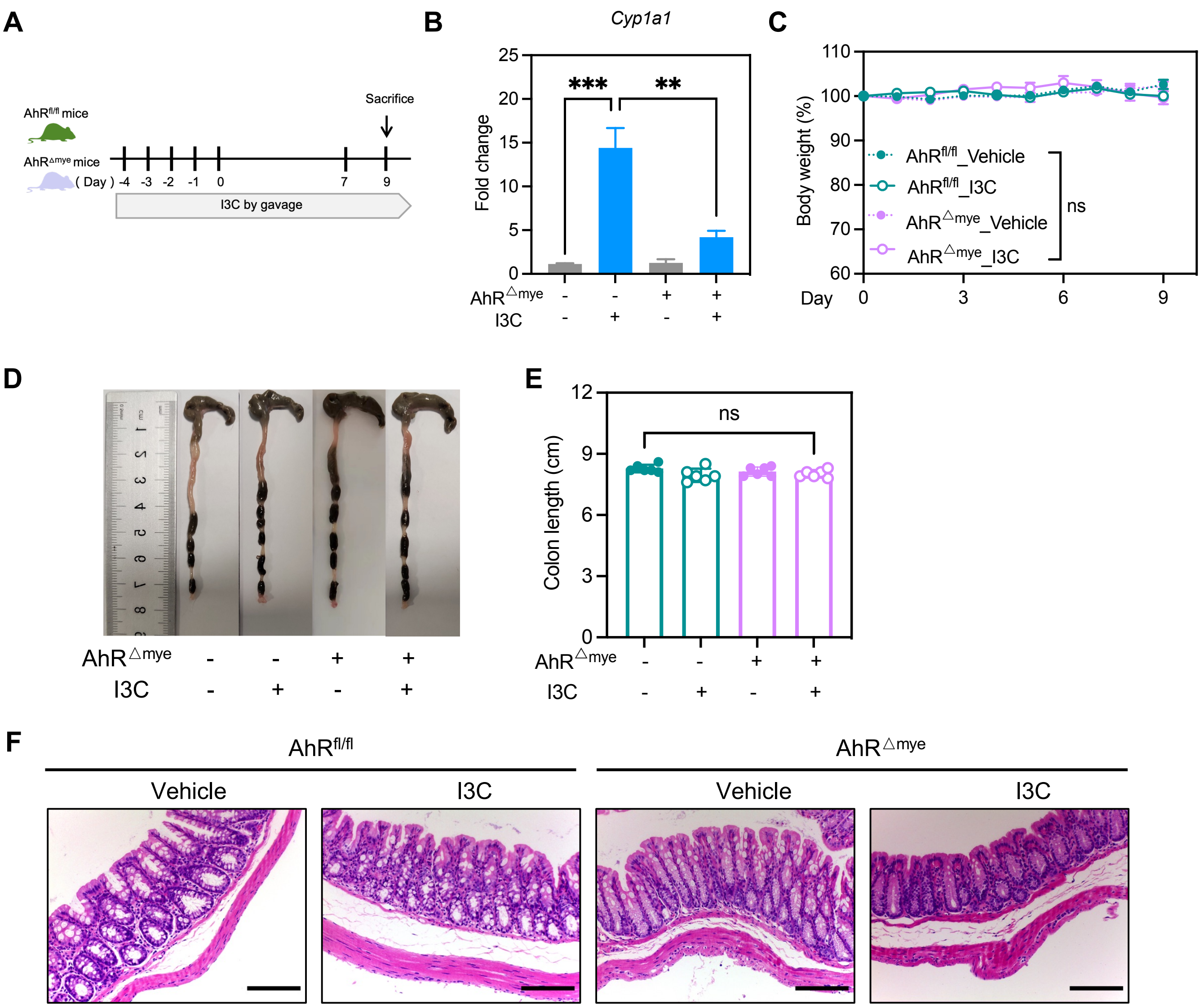

Figure S5. Pharmacologic administration of indole-3-carbinol (I3C) alone had no impact on body weight or colon morphology in AhR$^{\Delta Mye}$ and AhR$^{fl/fl}$ mice. (A) Schematic diagram of the timeline for I3C gavage. (B) mRNA expression levels of *Cyp1a1* in colon tissues quantified by RT-qPCR. (C) Body weight changes were monitored daily and are depicted as a percentage of initial body weight. (D) Representative morphology images of colons from each group. (E) Colon lengths were measured and recorded. (F) Representative histopathological images of H&E-stained colon sections. Scale bar, 200 µm. n = 6 per group. Statistical analysis of the data was performed using one-way ANOVA (B, E), and two-way ANOVA (C) followed by Tukey's multiple comparison tests. Data are shown as the mean ± SEM. ns, not significant; $^{**}P < 0.01$, $^{***}P < 0.001$. AhR, aryl hydrocarbon receptor; HE, hematoxylin and eosin; I3C, indole-3-carbinol; RT-qPCR, quantitative reverse transcription polymerase chain reaction.

Fig.S6

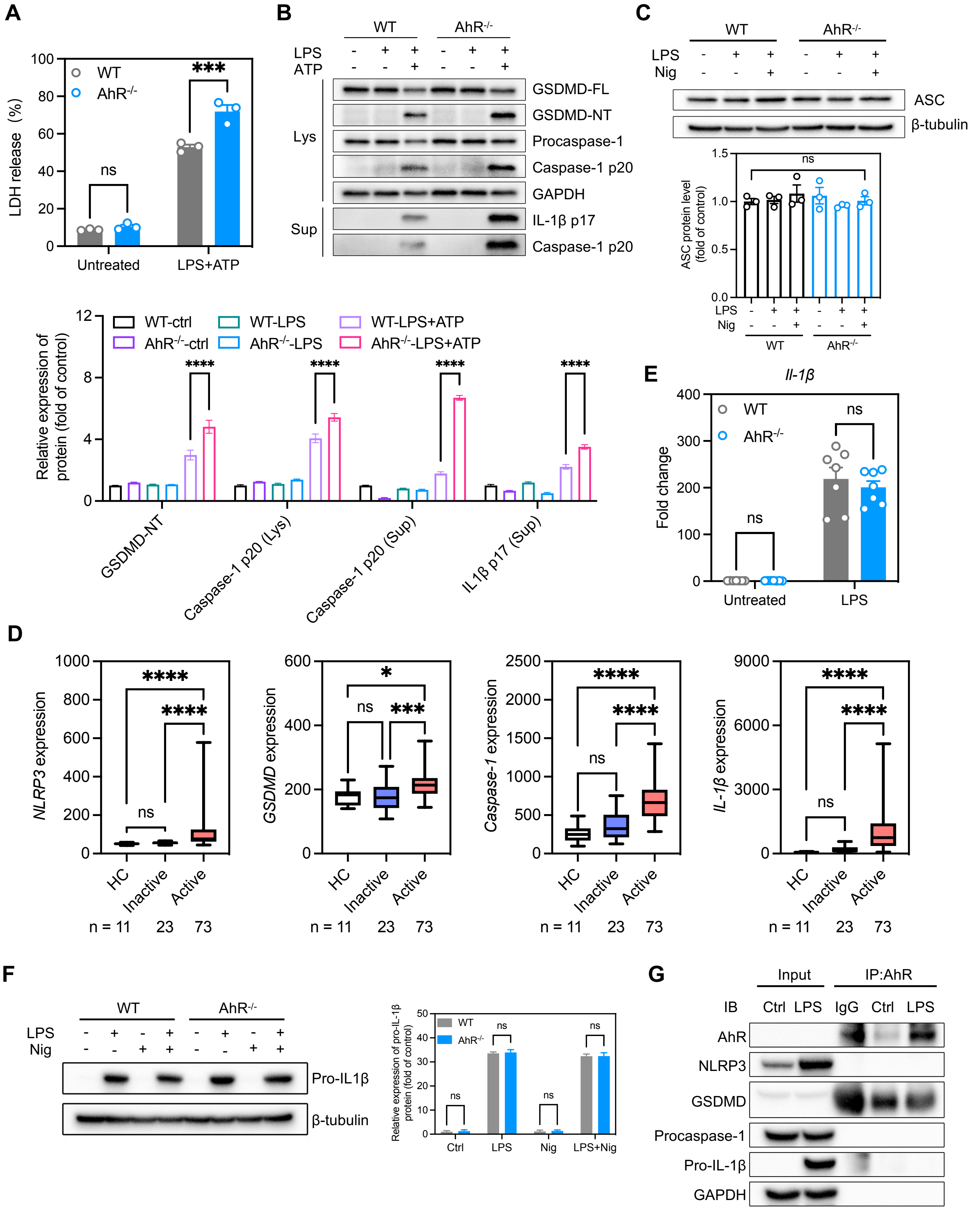

Figure S6. IL-1β expression did not differ significantly between WT and AhR$^{-/-}$ macrophages. BMDMs derived-from WT and AhR$^{-/-}$ mice were treated with LPS (200 ng/mL) for 4 h, followed by ATP (5 mM) or Nig (10 μM) for 30 min. (A) Cytotoxicity was detected by LDH release assay. (B) Proteins from cell lysates and supernatants were immunoblotted for full-length and cleaved caspase-1 and GSDMD, and mature IL-1β

(IL-1β p17). (C) ASC protein levels were determined by western blot analysis. (D) Database (GSE75214) analysis of *NLRP3*, *GSDMD*, *Caspase-1*, and *IL-1β* mRNA expression in intestinal mucosal biopsies from patients with UC and healthy controls. (E) *Il-1β* mRNA expression levels quantified by RT-qPCR. n = 7 per group. (F) Pro-IL1β protein levels determined by western blot analysis. (G) AhR and NLRP3 inflammasome component interaction in untreated and LPS-treated RAW264.7 cells were examined by immunoprecipitation assay and western blot analysis. Statistical analysis of the data was performed using one-way ANOVA (C) and two-way ANOVA (A, B, E, F) followed by either Tukey's or Sidak's multiple comparison tests, or Kruskal-Wallis test (D) followed by Dunn's multiple comparisons test. Data are shown as the mean ± SEM from three independent experiments. ns, not significant; *$P < 0.05$, *** $P < 0.001$, and **** $P < 0.0001$. AhR, aryl hydrocarbon receptor; ATP, adenosine triphosphate; ASC, apoptosis associated speck like protein containing a CARD; BMDMs, bone marrow derived macrophages; LDH, lactate dehydrogenase; Nig, nigericin; UC, ulcerative colitis.

Fig.S7

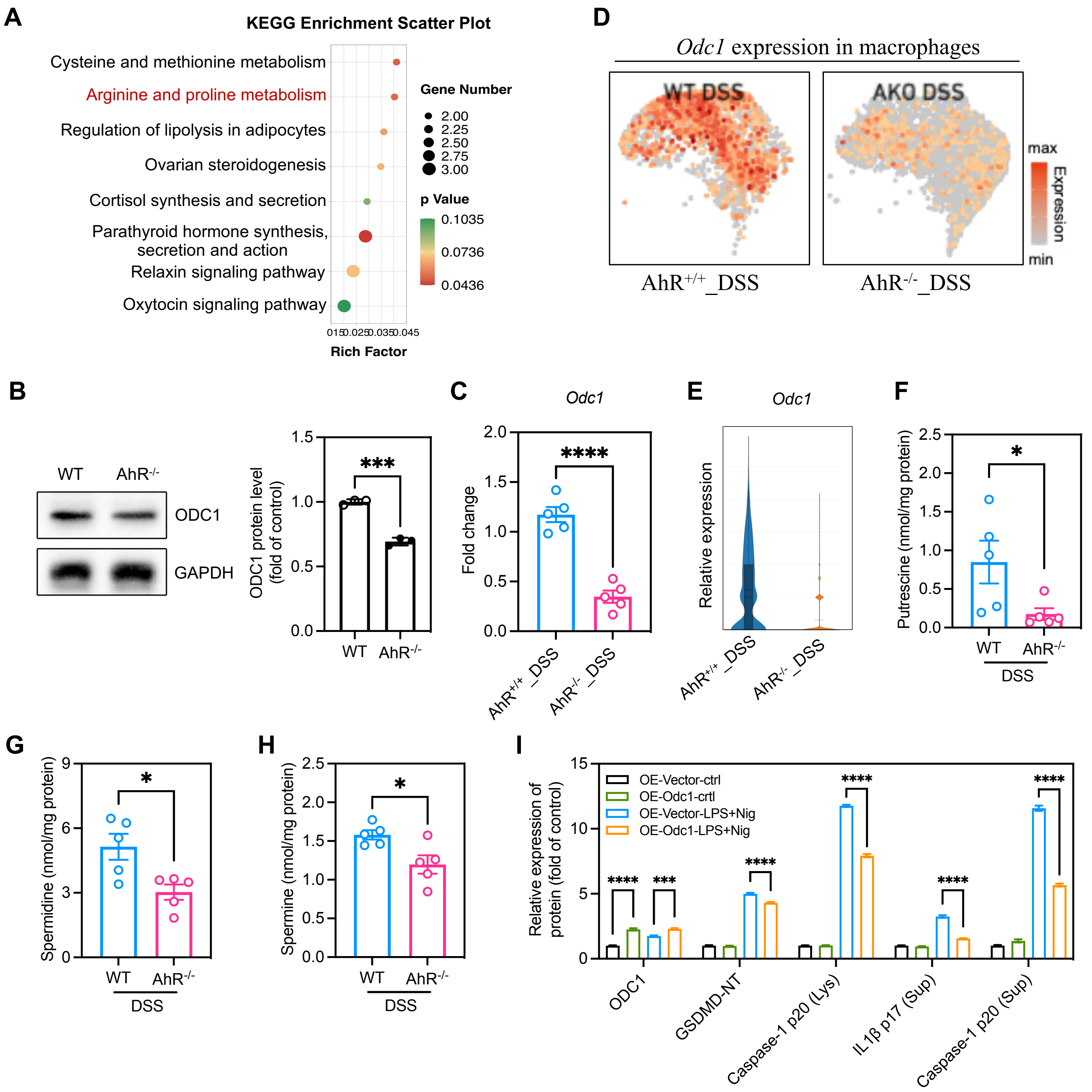

Figure S7. The relationship between AhR and Odc1 expression. (A) KEGG enrichment analysis showing the top eight pathways enriched in AhR-deficient BMDMs compared with WT BMDMs. (B) ODC1 protein levels were determined in WT and $AhR^{-/-}$ BMDMs. (C) *Odc1* mRNA levels in colon tissues from DSS treated $AhR^{-/-}$ mice and $AhR^{+/+}$ mice determined by RT-qPCR. (D) *Odc1* expression shown in the macrophage UMAP plots. Color intensity corresponds to the relative expression level of *Odc1*. (E) Violin plot comparing *Odc1* expression in colon macrophages between DSS treated $AhR^{-/-}$ mice and $AhR^{+/+}$ mice. (F-H) Colonic polyamine (putrescine, spermidine, spermine) levels were measured by liquid chromatography-mass spectrometry. (I) Quantitative protein analysis for Figure 6H. Statistical analysis of the data was performed using Mann Whitney test (F), two-tailed unpaired *t* test (B, C, G, H), and two-way ANOVA followed by Tukey's multiple comparison tests (I). Data are presented as the mean ± SEM. **P* < 0.05, ****P* < 0.001, and *****P* < 0.0001. AhR, aryl hydrocarbon receptor; BMDM, bone barrow derived macrophage; DSS: dextran sulfate sodium salt; KEGG, Kyoto Encyclopedia of Genes and Genomes; ODC1, ornithine decarboxylase 1; UMAP, uniform manifold approximation and projection.

Fig.S8

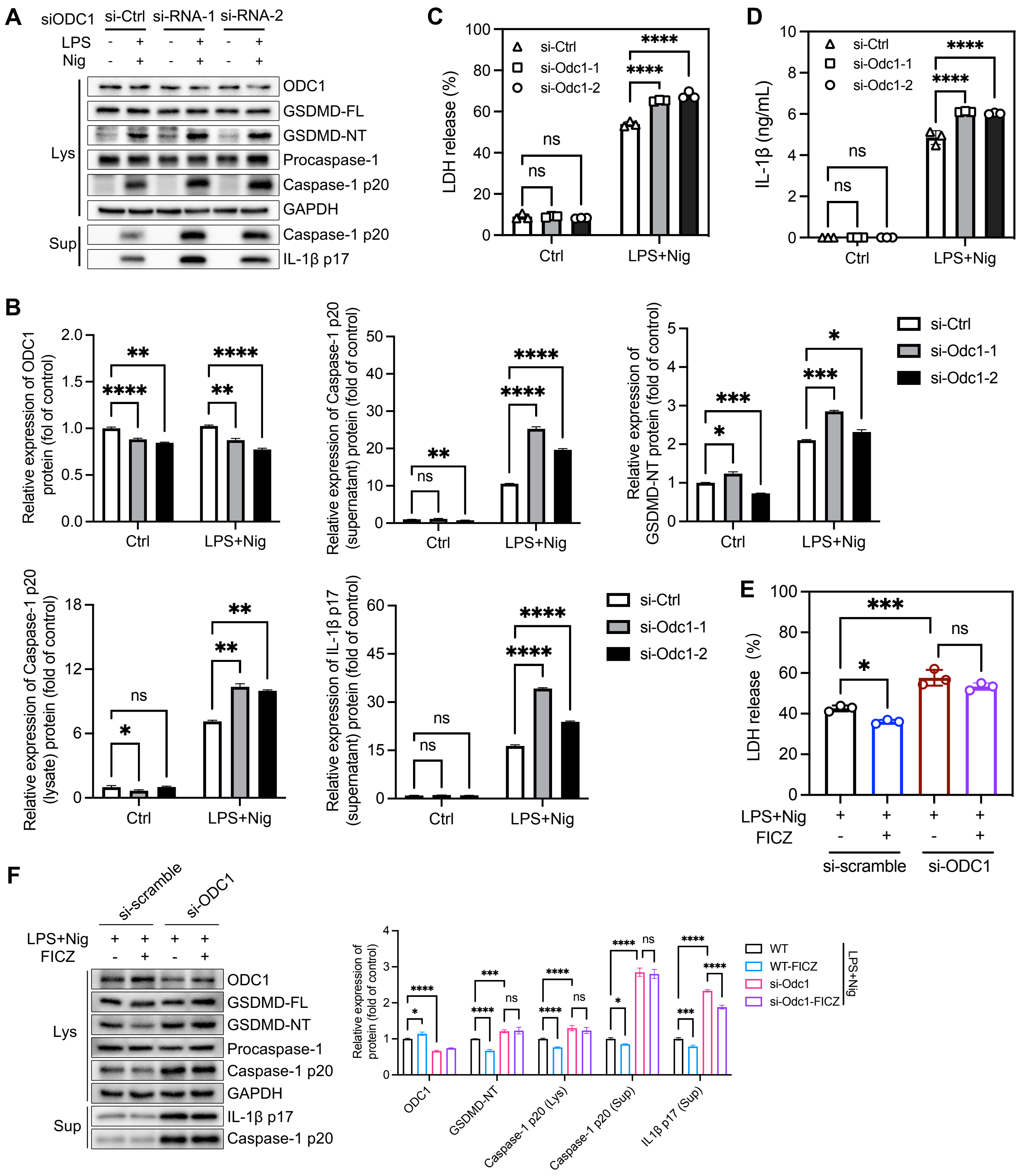

Figure S8. Knock-down of ODC1 promoted macrophage pyroptosis. (A) BMDMs derived from wild type mice were transfected with ODC1 siRNAs (200 nM), then stimulated with LPS (200 ng/mL) and Nig (10 μM) to induce pyroptosis. Proteins in cell lysates and supernatants were immunoblotted to detect ODC1, full-length and cleaved caspase-1 and GSDMD, and mature IL-1β (IL-1β p17). (B) Quantitative protein analysis for C. (C) Cytotoxicity was detected by LDH release assay. (D) IL-1β expression levels in cell culture supernatants determined by ELISA. (E) Cytotoxicity was assessed by LDH release. (F) Proteins from cell lysates and supernatants were immunoblotted for full-length and cleaved caspase-1 and GSDMD, and mature IL-1β (IL-1β p17). Statistical analysis of the data was performed using two-way ANOVA (B, C, D, F) or one-way ANOVA (E) followed by Tukey's multiple comparison tests. Data are shown as the mean ± SEM from three

independent experiments. ns, not significant; *$P < 0.05$, **$P < 0.01$, ***$P < 0.001$, and ****$P < 0.0001$. AhR, aryl hydrocarbon receptor; BMDMs, bone marrow derived macrophages; FICZ, 6-Formylindolo[3,2-b]carbazole; LDH, lactate dehydrogenase; Nig, nigericin; ODC1, ornithine decarboxylase 1.

Fig.S9

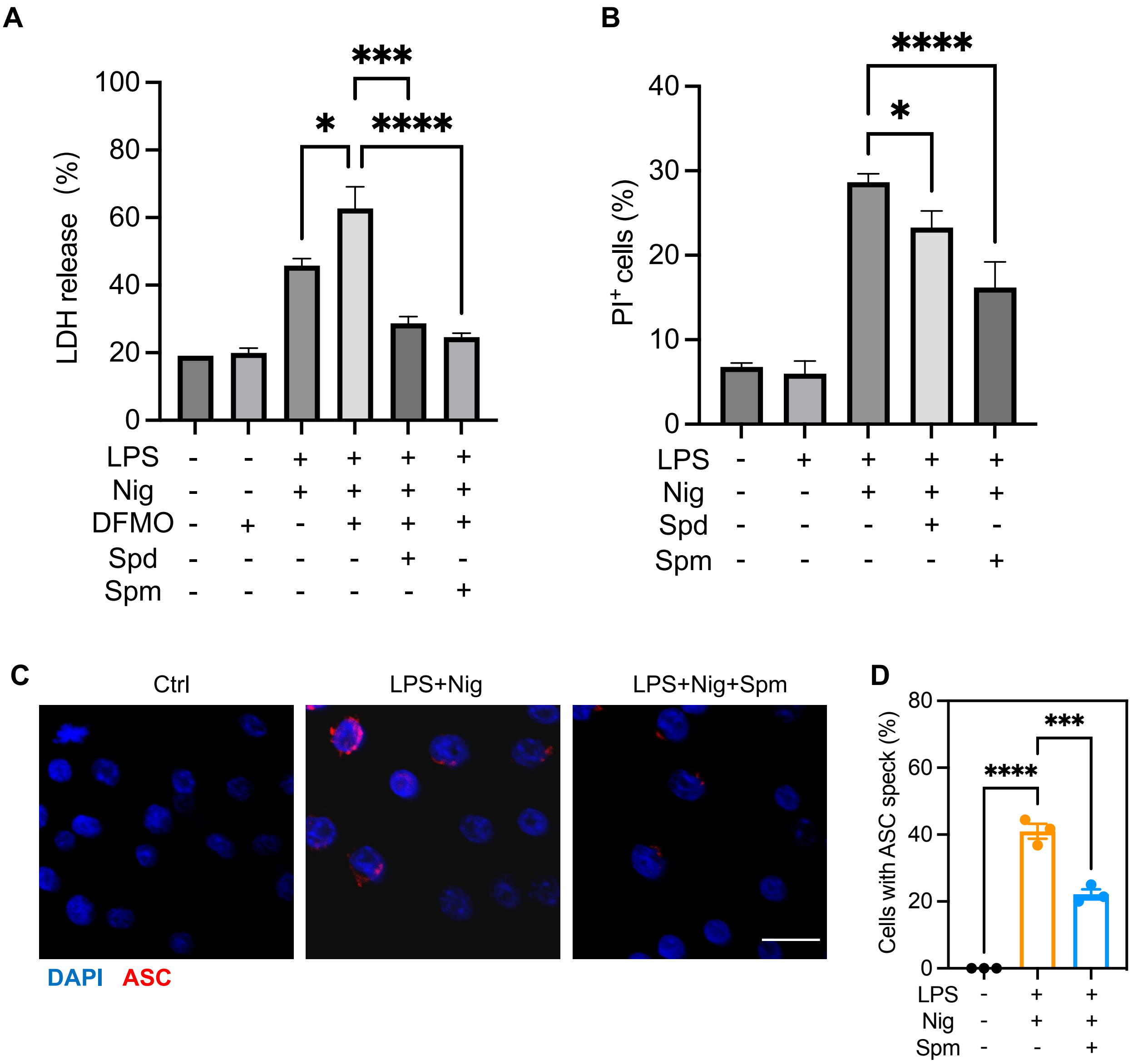

Figure S9. Spermidine and spermine inhibited macrophage pyroptosis. (A) BMDMs from wild type mice were treated with DFMO for 3 days; cell culture medium was replaced every day. Exogeneous Spd (50 µM) and Spm (50 µM) were added to the cell culture medium together with LPS, then cells stimulated with Nig to induce pyroptosis. Cell cytotoxicity was detected by LDH release assay. (B) Percentages of PI-positive cells were quantified by flow cytometry. n = 3 per group. (C) Representative immunofluorescence images of ASC speck formation (red). Nuclei were stained with DAPI (blue). Scale bar, 20 µm. (D) Bar chart showing percentages of cells containing a visible ASC speck. Statistical analysis of the data was performed using one-way ANOVA (A, B, D) followed by Tukey's multiple comparison tests. Data are shown as the mean ± SEM from three independent experiments. ns, not significant; *P < 0.05, ***P < 0.001, ****P < 0.0001. ASC, apoptosis associated speck like protein containing a CARD; BMDMs, bone marrow derived macrophages; DFMO, difluoromethylornithine; LDH, lactate dehydrogenase; Nig, nigericin; PI, propidium iodide; Spd, spermidine; Spm, spermine.

Fig.S10

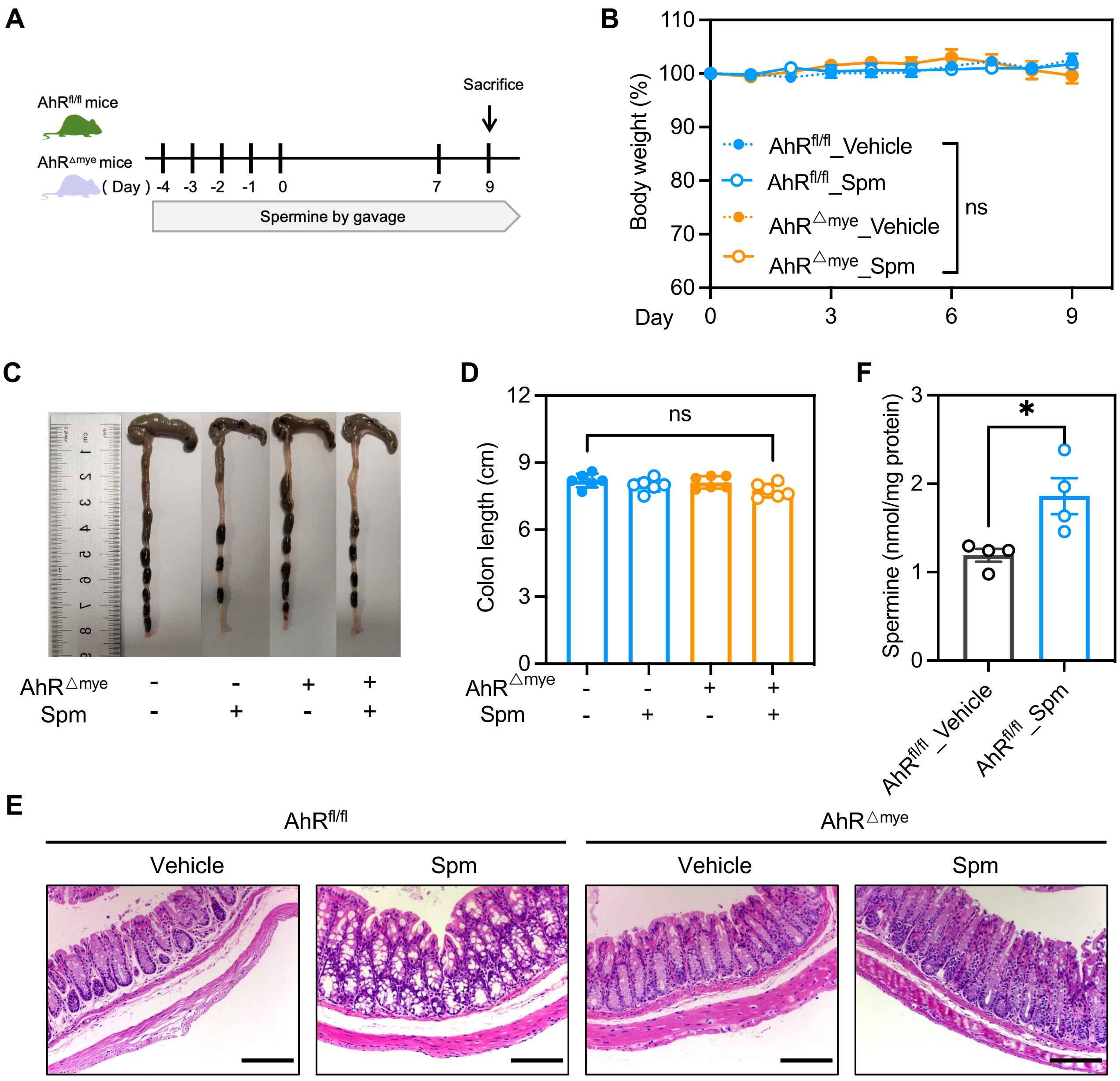

Figure S10. Pharmacologic administration of spermine had no impact on the body weight or colon morphology of AhR$^{\Delta Mye}$ and AhR$^{fl/fl}$ mice. (A) Schematic diagram of the timeline for Spm gavage. (B) Body weight changes were monitored daily and are depicted as the percentage of initial body weight. (C) Representative images of the morphology of colons from each group. (D) Colon lengths were measured and recorded. (E) Representative histopathological images of H&E-stained colon sections. Scale bar, 200 μm. n = 6 per group. (F) Colonic spermine levels were measured by liquid chromatography-mass spectrometry. Statistical analysis of the data was performed using two-tailed unpaired *t* test (F), one-way ANOVA (D), and two-way ANOVA (B) followed by Tukey's multiple comparison tests. Data are shown as the mean ± SEM. ns, not significant; **P* < 0.05. AhR, aryl hydrocarbon receptor; HE, hematoxylin and eosin; Spm, spermine.

Supplementary Tables

Table S1 Primer sequences to identify AhR$^{fl/fl}$Lyz2-Cre^{+} mice

Genes	Forward primer	Reverse primer
**Lyz2-Cre**	CTTGGGCTGCCAGAATTTCTC	CCCAGAAATGCCAGATTACG
		TTACAGTCGGCCAGGCTGAC
**AhR LoxP**	CAGTGGGAATAAGGCAAGAGT GA	GGTACAAGTGCACATGCCTGC

Table S2 The list of siRNA used in the study

Gene name	Sense:5’-3’	Anti-sense:5’-3’
**ODC1-Mus-1203**	GGCCAAACAUCUACUAUGUTT	ACAUAGUAGAUGUUUGGCCTT
**ODC1-Mus-264**	GUGCAAGCAAGACUGAAAUTT	AUUUCAGUCUUGCUUGCACTT
**ODC1-Mus-1104**	GCUGUAACCUGCCUGAAAUTT	AUUUCAGGCAGGUUACAGCTT

Table S3 Primer sequences for plasmids construct

Genes	Forward primer	Reverse primer
**pEGFP-C3-AhR**	AAGCTTATGAGCAGCGGCGCCAAC	CCG CGGTCAACTCTGCACCTTGCT
**pGL3-Basic-**	cgagctcttacgcgtgctagcCAGGATAACATT-	atgcagatcgcagatctcgagCTACTTAT-
**Odc1-promoter**	CTGGACATTGGCATTTT	ACGCCGAGCACGTCGTCA
**pcDNA3.1-ODC1**	CTTGGTACCGAGCTCGGATCCATGAGCA-	GAAGGGCCCTCTAGACTCGAGCTA
	GCTTTACTAAGGACGAGT	-CACATTGATCCTAGCAGAAG

Table S4 Primer sequences for RT-qPCR

Genes	Forward primer	Reverse primer
**Mus_β-actin**	CATTGCTGACAGGATGCAGAAGG	TGCTGGAAGGTGGACAGTGAGG
**Mus_IL-1β**	TGGACCTTCCAGGATGAGGACA	GTTCATCTCGGAGCCTGTAGTG
**Mus_NLRP3**	TCACAACTCGCCCAAGGAGGAA	AAGAGACCACGGCAGAAGCTAG
**Mus_ODC1**	TGCCACACTCAAAACCAGCAGG	ACACTGCCTGAACGAAGGTCTC
**Mus_Cyp1a1**	CATCACAGACAGCCTCATTGAGC	CTCCACGAGATAGCAGTTGTGAC

Table S5 Primer sequences used for ChIP analysis of Odc1 promoter

Genes	Forward primer	Reverse primer
**XRE-1**	GTGGCTGTCCACCACCTCTTACCT	GGCCACTCCGTGTTATGTTGGTGG
**XRE-2**	CAGCCAGGACTGGTGATGTGGTG	CACCCGCCCATACGCCCC
**XRE-3**	GCGTATGGGCGGGTGGGTG	GACTCCGTGGCGGCCAACC
